# Single-cell transcriptome conservation in cryopreserved cells and tissues

**DOI:** 10.1186/s13059-017-1171-9

**Published:** 2017-03-01

**Authors:** Amy Guillaumet-Adkins, Gustavo Rodríguez-Esteban, Elisabetta Mereu, Maria Mendez-Lago, Diego A. Jaitin, Alberto Villanueva, August Vidal, Alex Martinez-Marti, Enriqueta Felip, Ana Vivancos, Hadas Keren-Shaul, Simon Heath, Marta Gut, Ido Amit, Ivo Gut, Holger Heyn

**Affiliations:** 1 CNAG-CRG, Centre for Genomic Regulation (CRG), Barcelona Institute of Science and Technology (BIST), Barcelona, Spain; 2 Universitat Pompeu Fabra (UPF), Barcelona, Spain; 3 Department of Immunology, Weizmann Institute, Rehovot, Israel; 4 Chemoresistance and Predictive Factors Laboratory, Program Against Cancer Therapeutic Resistance (ProCURE), Catalan Institute of Oncology (ICO), Bellvitge Institute for Biomedical Research (IDIBELL), Barcelona, Spain; 5 Xenopat S.L., Business Bioincubator, Bellvitge Health Science Campus, Barcelona, Spain; 6 Department of Pathology, University Hospital of Bellvitge (IDIBELL), Barcelona, Spain; 7 Vall d’Hebron University Hospital, Barcelona, Spain; 8 Universitat Autònoma de Barcelona (UAB), Barcelona, Spain; 9 Vall d’Hebron Institute of Oncology (VHIO), Barcelona, Spain

**Keywords:** Single-cell genomics, RNA sequencing, Transcriptomics, MARS-Seq, Smart-seq2, Cryopreservation, Conservation, Peripheral blood mononuclear cells, PBMC, Patient-derived orthotopic xenograft, PDOX

## Abstract

A variety of single-cell RNA preparation procedures have been described. So far, protocols require fresh material, which hinders complex study designs. We describe a sample preservation method that maintains transcripts in viable single cells, allowing one to disconnect time and place of sampling from subsequent processing steps. We sequence single-cell transcriptomes from >1000 fresh and cryopreserved cells using 3'-end and full-length RNA preparation methods. Our results confirm that the conservation process did not alter transcriptional profiles. This substantially broadens the scope of applications in single-cell transcriptomics and could lead to a paradigm shift in future study designs.

**Electronic supplementary material:**

The online version of this article (doi:10.1186/s13059-017-1171-9) contains supplementary material, which is available to authorized users.

## Background

Within complex tissues, cells differ in the way their genomes are active. Despite the identical DNA sequence of single cells, their distinct interpretation of the genetic sequence makes them unique and defines their phenotype [1]. While in many complex biological systems cell-type heterogeneity has been extensively analyzed in molecular and functional experiments, its extent could only be estimated due to the technical limitation to assess the full spectrum of variability. With the advent of single-cell genomics, cell-type composition can be deconvoluted for unprecedented insights into the complexity of multicellular systems. Single-cell transcriptomics studies resolved the neuronal heterogeneity of the retina [2], the cortex, and the hippocampus [3, 4], but also advanced our definition of hematopoietic cell states [5, 6]. Moreover, single-cell genomics studies shed light on cellular relationships in dynamic processes, such as embryo development [7] and stem cell differentiation [8]. The assessment of hundreds to thousands of single-cell gene expression signatures allowed tissue decomposition at ultra-high resolution. In addition to providing insights into the complexity of the analyzed samples, single-cell studies provide an invaluable resource of biomarkers that define cell types [3, 9] or differentiation states [10].

Different single-cell RNA sequencing (RNA-seq) techniques allow the quantification of minute transcript amounts from up to thousands of single cells; however, their exclusive dependence on fresh starting material strongly restricts study designs [11]. In particular, the need for immediate sample processing hindered complex study setups, such as time course studies or sampling at locations without access to single-cell separation devices. Seminal work on the composition of complex systems was performed with readily accessible tissues from model organisms and the extent to which conclusions can be projected to human physiology is limited [2, 3, 5].

Here we evaluate a sample cryopreservation method that allows disconnecting time and location of sampling from subsequent single-cell processing steps. It enables complex experimental designs and widens the scope of accessible specimens. We demonstrated that cryopreservation maintains cellular structures and integrity of RNA molecules for single-cell separation months after archiving by analyzing 1486 single-cell transcriptomes from fresh or cryopreserved cells from cell lines or primary tissues.

## Results and discussion

Cell integrity and RNA quality present crucial requirements for successful single-cell transcriptome sequencing experiments. Conventional conservation processes, such as freezing, lead to crystallization and disruption of cellular membranes, which impedes subsequent single-cell preparation. To conserve intact and viable cells for cell and tissue archiving, cryoprotectants are commonly used; however, their compatibility with single-cell experiments has not been established. We tested whether cells preserved with the cryoprotectant dimethyl-sulfoxide (DMSO) are suitable for single-cell genomics workflows. We sequenced 670 fresh and 816 cryopreserved single cells derived from cell lines and primary tissues (Additional file 1: Figure S1 and Additional file 2: Table S1). Single-cell transcriptome libraries were prepared with the massively parallel single-cell RNA-sequencing (MARS-Seq) protocol [5, 6]. To evaluate the impact of cryopreservation on single-cell full-length transcriptomes, we applied the Smart-seq2 protocol [12]. A variety of statistical methods, including the most common measures in single-cell genomics, were applied.

**Fig. 1:**
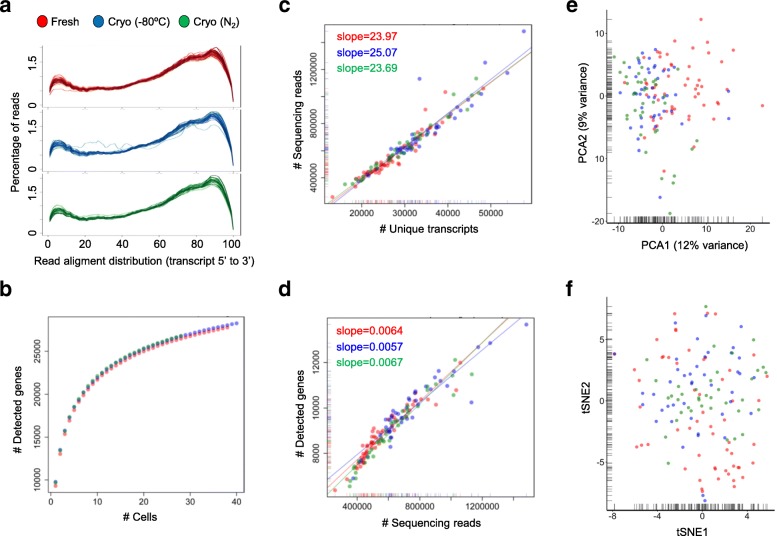
Comparative analyses of MARS-Seq-derived single-cell transcriptome data from fresh (*red*) and cryopreserved (–80 °C: *blue*; liquid nitrogen: *green*) HEK293 cells. **a** *Mapping distribution* of sequencing reads after MARS-seq library preparation. Each *line* represents a single cell and transcript sizes are scaled from 0 to 100. **b** Cumulative gene counts split by fresh and cryopreserved cells and analyzed using randomly sampled cells (average of 100 permutations). **c**, **d** Comparative analysis of the number of sequencing reads and detected transcripts (**c**) or genes (**d**) per cell using a linear model. The slope of the regression line was calculated separately for fresh and cryopreserved cells. **e**, **f** Gene expression profile variances between fresh and cryopreserved cells displayed as principal component analysis (PCA, **e**) or as t-distributed stochastic neighbor embedding (t-SNE) representation (**f**) using the 100 most variable genes

We used the MARS-Seq sample preparation protocol to determine potential impacts of the cryopreservation procedure on single-cell RNA profiles. We initially isolated single cells from four cell lines HEK293 (human embryonic kidney cells), K562 (human leukemia cells), NIH3T3 (mouse embryo fibroblasts), and MDCK (canine adult kidney cells) by fluorescence-activated cell sorting (FACS). The cells were either freshly harvested or cryopreserved in the presence of DMSO at –80 °C or in liquid nitrogen prior to single-cell separation and library preparation. To minimize technically introduced batch effects between conditions, all single cells were processed simultaneously for library preparations and sequencing reactions. As expected, the freezing process resulted in an elevated proportion of damaged cells, indicated by the positive staining with propidium iodide. HEK293, K562, and NIH3T3 presented 14%, 2%, and 15% of damaged cells when processed freshly, 66%, 55%, and 20% when cryopreserved at –80 °C, respectively. Conservation in liquid nitrogen slightly improved cell viability showing 61%, 49%, and 17% of damaged cells, respectively. Nevertheless, sequencing reads produced from sorted viable cells displayed an equal distribution over the transcripts (characteristic 3’ bias for MARS-Seq libraries), excluding systematic errors in the library preparation process (Fig. 1a).

Following gene expression quantification, we evaluated to which extent transcriptome information is maintained within single cells and compared transcript and gene information content between fresh and the cryopreserved (–80 °C and liquid nitrogen) conditions. A comparable number of genes was detected by cumulating information from single cells, suggesting that the power to detect gene transcripts in the conserved material is not reduced (Fig. 1b and Additional file 1: Figure S2). We further observed that libraries from fresh and cryopreserved cells produced a similar number of sequencing reads (Additional file 1: Figure S3). Importantly, we found a highly correlated linear relationship between the number of sequencing reads and unique transcripts for both conditions. This indicates that the capacity to capture transcript molecules and the library complexity is not different between both conditions (linear regression model; Fig. 1c and Additional file 1: Figure S4). In line, equal sequencing depth identified similar numbers of expressed genes (linear regression model; Fig. 1d and Additional file 1: Figure S5).

We further assessed the impact of sample conservation on single-cell transcriptome profiles. Genes with variable expression patterns are commonly utilized for the identification of cell subtypes, thus differences between conditions could introduce technical artefacts that complicate data interpretation. Importantly, dimensionality reduction representations using the most variable genes (MVG) point to a general conservation of the single-cell transcriptome during cryopreservation. Expression patterns from cryopreserved cells were similar to freshly processed cells in principal component analyses (PCA) (Fig. 1e and Additional file 1: Figure S6) and t-distributed stochastic neighbor embedding representations (t-SNE) (Fig. 1f and Additional file 1: Figure S6). Small differences between fresh and cryopreserved samples (Fig. 1e and Additional file 1: Figure S6) were considerably lower than technically introduced batch effects when two sequencing pools were compared (Additional file 1: Figure S7a, b) and could be the result of different sampling time points (biological variability). The homogeneity between single cells and conditions in t-SNE representations was stable with varying perplexity parameter selection, underlining the robustness of the results (Additional file 1: Figure S8). Determining the MVG separately for fresh and cryopreserved samples showed an average overlap of 53% (range 51–56%). Randomly subsampling (100 permutations) only fresh cells into two groups resulted in an average overlap of 38% (range 37–41%), while MVG overlapped in 36% (range 35–37%) when fresh and cryopreserved cells were sampled at the same cell numbers. Analyzing transcriptional uniformity across cell types, variably expressed genes distinguished between K562 and HEK293 cells, while processing conditions mixed homogenously in dimensionality reduction representations (Additional file 1: Figure S9a, b).

**Fig. 2:**
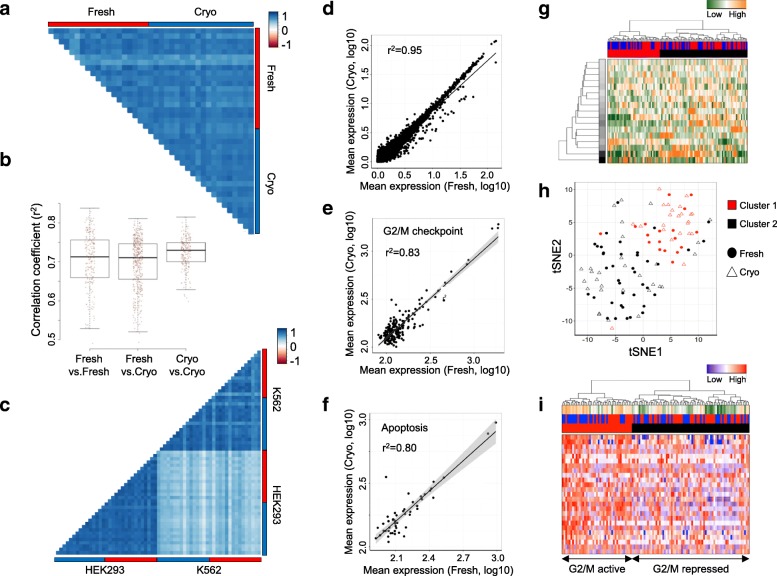
Correlating MARS-Seq-derived single-cell transcriptomes from fresh (*red*) and cryopreserved (*blue*) HEK293 cells identify subpopulations. **a** Pearson’s correlation analysis between 20 randomly selected fresh and cryopreserved cells displaying the correlation coefficient (r^2^). **b** Distribution of Pearson’s correlation coefficients (r^2^) within and between processing conditions. The median coefficients are indicated. **c** Pearson’s correlation analysis between 20 randomly selected fresh and cryopreserved HEK293 or K562 cells displaying the correlation coefficient (r^2^). **d**–**f** Linear regression model comparing average gene expression levels of (**d**) expressed, (**e**) cell cycle (G2/M checkpoint, and (**f**) apoptosis genes. The coefficient of determination (r^2^) is indicated. **g** Hierarchical clustering of single cells based on transcriptional programs (defined by Gene Ontology) and correlating gene sets [21]. Transcriptional programs and gene clusters are summarized in aspects. Displayed are the most variable aspects (*rows*) and their importance (*row colors*). Cells are assigned to condition (fresh: *red*; cryopreserved: *blue*) and clusters. **h** A t-SNE representation of similarities between cells using previous defined distances and cluster identity (as in **g**). Conditions are indicated (fresh: *circle*; cryopreserved: *triangle*). **i** Hierarchical cluster of single cells (as in **g**) displaying the 25 most variable cell cycle genes (G2/M checkpoint). Expression levels of the cell cycle signature are summarized (first panel; high: *orange*, low: *green*) and conditions (second panel; fresh: *red*; cryopreserved: *blue*) and clusters are indicated

High similarities between single cells from fresh and cryopreserved (–80 °C) cells were confirmed by direct correlation analysis, showing highly consistent and representative gene expression profiles of HEK293 cells after cell conservation (Fig. 2a). As expected analyzing homogenous cell populations, expression profiles showed high correlation values between single cells of the same type and condition (Pearson’s correlation test, Fig. 2b). However, also between conditions, transcription profiles were highly correlated (Pearson’s correlation test, Fig. 2a, b), suggesting the freezing process to conserve single-cell transcriptome profiles. These results were reproducible across the different cell types and species (Additional file 1: Figure S10a–h). Further, we evaluated the specificity of such analysis by combining the analysis of different cell types. In accordance with the presence of tissue-specific expression programs, HEK293 and K562 cells displayed correlating profiles of their respective single cells and highly decreased associations across samples (Fig. 2c). These patterns were conserved in fresh and cryopreserved cells. Consistent expression profiles were further supported by highly correlating mean expression values when directly comparing both conditions (Fig. 2d and Additional file 1: Figure S10i–k).

In order to evaluate potential impacts on comparative expression analyses involving fresh and conserved sample types, we assessed differentially expressed genes between both conditions. In the four-cell line, we only detected a single significantly differentially expressed gene between fresh and cryopreserved samples (adjusted *p* value < 0.01, Additional file 2: Tables S2–5), supporting the possibility to include conserved material in studies profiling freshly processed samples. Finally, biological processes that one might suspect to change due to a challenge, such as cell cycle and apoptotic programs, remained unchanged (Fig. 2e, f). Moreover, cell subpopulations identified by hierarchical clustering of the most variable gene sets (see “Material and methods”) were equally identified in fresh and cryopreserved samples (Fig. 2g, h). We detected a similar proportion of fresh and conserved cells in a subpopulation with an activated cell cycle program indicated by G2/M checkpoint genes (χ^2^ test, *p* = 0.83; Fig. 2i). Of note, comparable compositions of cellular subtypes were also identified processing fresh and conserved cells separately, with 37% and 34% of cells pointing to cell cycle activation, respectively (Additional file 1: Figure S11).

**Fig. 3:**
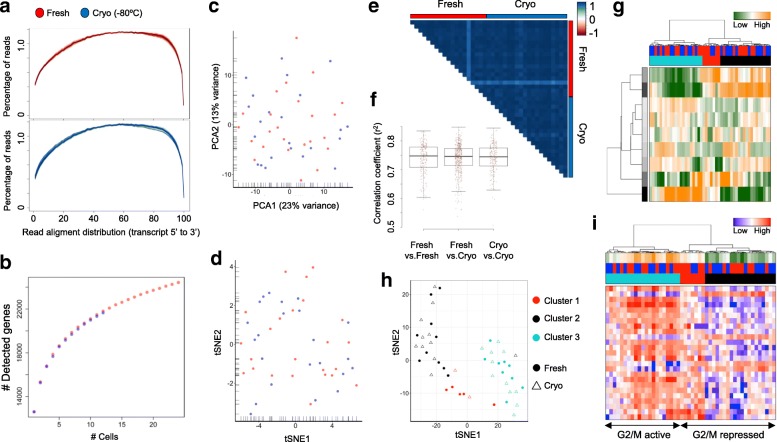
Comparative analyses of Smart-seq2-derived single-cell transcriptomes from fresh (*red*) and cryopreserved (*blue*) HEK293 cells. **a** Sequencing read distribution following RNA library preparation of full-length transcripts. Each line represents a single cell and transcript sizes are scaled from 0 to 100. **b** Cumulative gene counts split by fresh and cryopreserved cells and analyzed using randomly sampled cells (average of 100 permutations). **c**, **d** Gene expression variances of single cells displayed as PCA (**c**) or t-SNE representation (**d**) using the 100 most variable genes. **e** Pearson’s correlation analysis between 20 randomly selected fresh and cryopreserved cells displaying the correlation coefficient (r^2^). **f** Distribution of Pearson’s correlation coefficients (r^2^) within and between processing conditions. The median coefficients are indicated. **g** Hierarchical clustering of single cells based on transcriptional programs (defined by Gene Ontology) and correlating gene sets [21]. Transcriptional programs and gene clusters are summarized in aspects. Displayed are the most variable aspects (*rows*) and their importance (*row colors*). Cells are assigned to conditions (fresh: *red*; cryopreserved: *blue*) and clusters. **h** A t-SNE representation of similarities between cells using previous defined distances and cluster identities (as in **g**). Conditions are indicated (fresh: *circle*; cryopreserved: *triangle*). **i** Hierarchical clustering (as in **g**) displaying the 25 most variable cell cycle genes (G2/M checkpoint). Expression levels of the cell cycle signature are summarized (first panel; high: *orange*, low: *green*) and conditions (second panel; fresh: *red*; cryopreserved: *blue*) and clusters are indicated

**Fig. 4:**
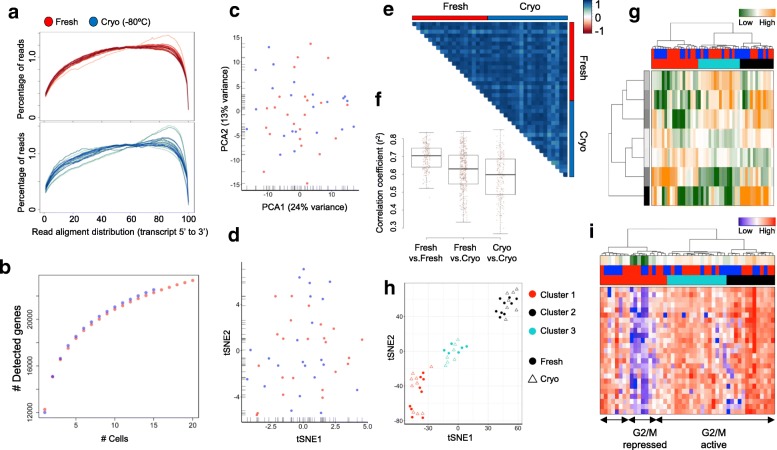
Comparative analyses of Smart-seq2-derived single-cell transcriptomes from fresh (*red*) and cryopreserved (*blue*) K562 cells. **a** Sequencing read distribution following library preparation of full-length transcripts. Each line represents a single cell and transcript sizes are scaled from 0 to 100. **b** Cumulative gene counts split by fresh and cryopreserved cells and analyzed using randomly sampled cells (average of 100 permutations). **c**, **d** Gene expression variances between single cells displayed as PCA (**c**) or t-SNE representation (**d**) using the 100 most variable genes. **e** Pearson’s correlation analysis between 20 randomly selected fresh and cryopreserved cells displaying the correlation coefficient (r^2^). **f** Distribution of Pearson’s correlation coefficients (r^2^) within and between processing conditions. The median coefficients are indicated. **g** Hierarchical clustering of single cells based on transcriptional programs (defined by Gene Ontology) and correlating gene sets [21]. Transcriptional programs and gene clusters are summarized in aspects. Displayed are the most variable aspects (*rows*) and their importance (*row colors*). Cells are assigned to conditions (fresh: *red*; cryopreserved: *blue*) and clusters. **h** A t-SNE representation of similarities between cells using previous defined distances and cluster identities (as in **g**). Conditions are indicated (fresh: *circle*; cryopreserved: *triangle*). **i** Hierarchical clustering (as in **g**) displaying the 25 most variable cell cycle genes (G2/M checkpoint). Expression levels of the cell cycle signature are summarized (first panel; high: *orange*, low: *green*) and conditions (second panel; fresh: *red*; cryopreserved: *blue*) and clusters are indicated

We extended the results studying single-cell libraries produced using the Smart-seq2 protocol, which is a widely used technique to sequence full-length transcripts from single cells [12]. To this end, we produced Smart-seq2 libraries for 48 fresh and 47 cryopreserved (–80 °C) HEK293 and K562 cells, respectively, and sequenced to an average depth of 7.7 million reads per single cell (Additional file 1: Figure S1). No systematic bias in sequencing read distribution across the transcripts was detected, supporting a conserved integrity of the RNA following cryopreservation (Figs. 3a and 4a). Most libraries showed an equal distribution of sequencing reads from the 5’- to the 3´-end, indicating the detection of full-length transcripts from both conditions. Of note, while HEK293-derived libraries presented extremely consistent distribution profiles between cells and conditions (Fig. 3a), K562 cells displayed higher heterogeneity (Fig. 4a). Here, a few libraries observed in fresh and cryopreserved samples showed a bias towards the 3’-end, pointing to a partial degradation of the RNA. We excluded that the increased coverage heterogeneity observed for the cryopreserved K562 cells (Fig. 4a) is indicative of the cryopreservation performance by sequencing an additional sample with Smart-seq2. Here, the analysis of a patient-derived orthotopic xenograft (PDOX) of a lung adenocarcinoma cryopreserved for six months did not show an elevated heterogeneity between samples across the transcripts (Additional file 1: Figure S12a).

Cumulating gene expression information over single cells pointed to conserved transcriptome content in archived samples (Figs. 3b and 4b). Further, dimensionality reduction representation of the most variable genes could not distinguish between fresh and cryopreserved cells (Figs. 3c, d and 4c, d) and clearly separated the analyzed tissue types (Additional file 1: Figure S12b, c). Correlation analysis of gene expression profiles from single cells supported transcriptional profiles to be highly conserved following cryopreservation (Figs. 3e, f and 4e, f). Hierarchical clustering and t-SNE representation of the most variable gene sets (see “Material and methods”) were able to identify subpopulations in HEK293 (Fig. 3g, h) and K562 (Fig. 4g, h) samples and did not point to proportional differences between conditions (χ^2^ test, *p* = 0.46 and *p* = 0.86, respectively). Consistent with cell populations identified using MARS-Seq, 44% of HEK293 cells presented an activated G2/M checkpoint program (Fig. 3i), a result that could be replicated using separate analysis for fresh and frozen samples (Additional file 1: Figure S13a, b). Finally, only two genes were detected to be differentially expressed between both conditions in HEK293 cells (adjusted *p* value < 0.01, Additional file 2: Table S6), further supporting the possibility to join fresh and conserved samples in combined studies. Of note, significantly differentially expressed genes showed high variance within the conditions, supporting the possibility that the low number of analyzed cells led their identification and not differences between conditions per se (Additional file 1: Figure S13c). Interestingly, we identified a small subpopulation of K562 cells with a repressed G2/M checkpoint program (Fig. 4i), a result that could be replicated in separate analysis of fresh and cryopreserved cells (Additional file 1: Figure S14a, b). Comparing both conditions in the K562, we detected ten genes to be differentially expressed (adjusted *p* value < 0.01, Additional file 2: Table S7), which again presented high variability within the respective conditions (Additional file 1: Figure S14c).

Although conserving cell cultures for single-cell analysis opens up the applicability to more complex experimental designs, we intended to further widen the application spectrum to complex primary tissues. We performed MARS-Seq experiments on fresh and cryopreserved human peripheral blood mononuclear cells (PBMC), mouse colon tissue, and finally extended the work to human tumor samples.

**Fig. 5:**
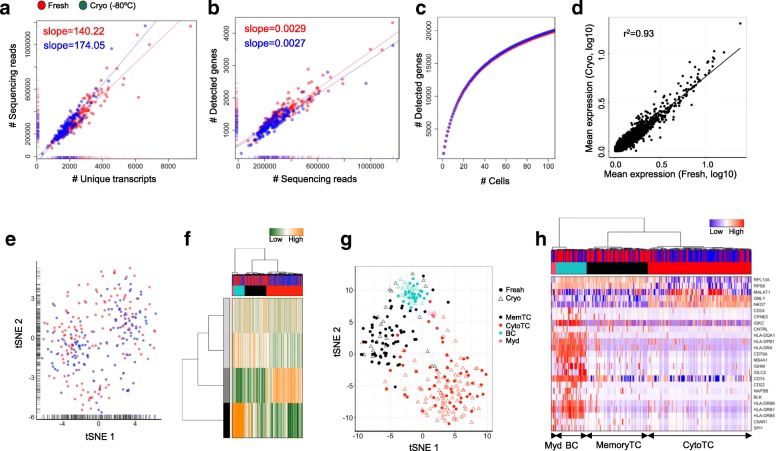
Correlating single-cell transcriptome data from fresh (*red*) and cryopreserved (*blue*) samples determines cell subtypes in PBMC. **a**, **b** Comparative analysis of the number of sequencing reads and detected transcripts (**a**) or genes (**b**) per cell using a linear model. The slope of the regression line was calculated separately for fresh and cryopreserved cells. **c** Cumulative gene counts split by fresh and cryopreserved cells and analyzed using randomly sampled cells (average of 100 permutations). **d** Linear regression model comparing average gene expression levels of expressed genes. The coefficient of determination (r^2^) is indicated. **e** Gene expression variances displayed as t-SNE representation using the 100 most variable genes. **f** Hierarchical clustering of single cells based on transcriptional programs (see “Material and methods”) and correlating gene sets [21]. Transcriptional programs and gene clusters are summarized in aspects. Displayed are the most variable aspects (*rows*) and their importance (*row colors*). Cells are assigned to condition (fresh: *red*; cryopreserved: *blue*) and clusters. **g** A t-SNE representation of similarities between cells using distances and cluster identities (as in **f**). Conditions are indicated (fresh: *circle*; cryopreserved: *triangle*). Cell types were annotated based on marker gene expression (*BC* B-cells, *CytoTC* cytotoxic T-cells, *MemTC* memory T-cells, *Myd* myeloid cells). **h** Hierarchical clustering of single cells (as in **f**). Displayed are the expression levels of the 25 most variable genes implicated in cluster formation. Cells are assigned to conditions (first panel: fresh: *red*; cryopreserved: *blue*) and clusters

We prepared MARS-Seq libraries for 341 cells derived from PBMC. While the freshly prepared sample did not show any sign of damaged cells, 23% of cryopreserved PBMCs stained positive with the marker reagent propidium iodide. Consistent with the results obtained from the cell line experiments, fresh and cryopreserved blood cells produced libraries of comparable complexity. We found a similar linear relationship between the number of sequencing reads and unique transcript counts (Fig. 5a) or the number of detected genes (Fig. 5b), suggesting equal transcriptome capture efficiencies in for both conditions. In line, cumulating gene information over single cells revealed highly comparable numbers of expressed genes in both datasets (Fig. 5c). Correlation analysis of average gene expression levels between both conditions further supported an efficient transcriptome conservation following cryopreservation (Fig. 5d). Dimensionality reduction representation of the most variable genes across datasets clearly suggested gene expression profiles to be unaltered following cell conservation (Fig. 5e). In line, we could not detect differentially expressed genes between fresh and cryopreserved single cells (Additional file 2: Table S8).

Mononuclear blood cells consist of a variety of well-defined subtypes, with marker genes indicating distinct cell populations. Consequently, we performed sample deconvolution to identify and assign blood subpopulations. Using clustering of correlating gene sets and signatures defined through the analysis of sorted blood cell types (see “Material and methods”), we observed four distinct cell populations, both detectable in fresh and cryopreserved samples (Fig. 5f–h). Based on marker genes and cell-type signatures, we assigned the four subpopulations to represent cytotoxic T-cells (expression of NKG7/GNLY/GZMB, red cluster, Additional file 1: Figure S15a), memory T-cells (expression of CD3D/G/E and CD8A/B, black cluster, Additional file 1: Figure S15b), B-cells (expression of CD24 and CD79A/B, turquois cluster, Additional file 1: Figure S15c), and myeloid cells (expression of CD33, pink cluster, Additional file 1: Figure S15d). Due to the limited number of cells analyzed, we were not able to distinguish between CD4-positive and CD8-positive cell populations or to clearly define natural killer cells within the cytotoxic subpopulation. Surprisingly, although the B-cell, monocyte and T-cell clusters were formed by equal proportions of fresh and cryopreserved cells (χ^2^ test, *p* = 0.28; Fig. 5f), we detected a bias towards preserved T-cells in the cytotoxic subpopulation (χ^2^ test, *p* = 0.0005; Fig. 5f). While this bias could represent donor blood composition variation at the sampling time points, we cannot finally exclude that it points to a technical artefact introduced by the preservation process and further tests are required with a focus on specific blood cell populations. Nevertheless, all main cell-type clusters could be detected at equal proportions using fresh and cryopreserved samples (Fig. 5f), suggesting that cryopreserved blood could be a suitable resource for single-cell transcriptomics analysis.

**Fig. 6:**
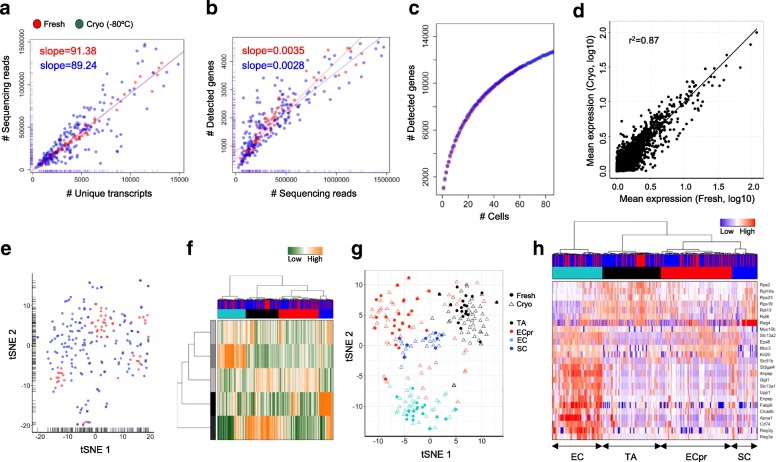
Single-cell transcriptome data from fresh (*red*) and cryopreserved (*blue*) mouse colon cells. **a**, **b** Comparative analysis of the number of sequencing reads and detected transcripts (**a**) or genes (**b**) using a linear model. The slope of the regression line was calculated separately for fresh and cryopreserved cells. **c** Cumulative gene counts split by fresh and cryopreserved cells and analyzed using randomly sampled cells (average of 100 permutations). **d** Linear regression model comparing average gene expression levels of expressed genes. The coefficient of determination (r^2^) is indicated. **e** Gene expression variances displayed as t-SNE representation using the 100 most variable genes. **f** Hierarchical clustering of single cells based on transcriptional programs (defined by Gene Ontology) and correlating gene sets [21]. Transcriptional programs and gene clusters are summarized in aspects. Displayed are the most variable aspects (*rows*) and their importance (*row colors*). Cells are assigned to condition (fresh: *red*; cryopreserved: *blue*) and clusters. **g** A t-SNE representation of similarities between cells using distances and cluster identities (as in **f**). Conditions are indicated (fresh: *circle*; cryopreserved: *triangle*). Cell types were annotated based on marker gene expression [9] (*TA* transit amplifying, *ECpr* enterocytes precursors, *EC* enterocytes, *SC* secretory cells). **h** Hierarchical clustering of single cells (as in **f**). Displayed are the expression levels of the 25 most variable genes implicated in cluster formation. Cells are assigned to condition (first panel: fresh: *red*; cryopreserved: *blue*) and clusters

A fresh mouse colon sample was split and one part was cryopreserved for one week before single-cell separation. As observed previously, cryopreservation resulted in an increased proportion of damages cell, detecting 30% in fresh and 68–71% in conserved samples. This proportion was similar comparing samples cryopreserved as minced tissue (68%) or as a single-cell solution (71%). Due to the donor-matched design, fresh and cryopreserved cells could not be processed in the same library and sequencing pools, resulting in confounding batch effects when directly comparing both conditions. However, highly similar library complexity and an unaltered power to detect gene signatures and cell types further supported the value of cryopreserved tissue in single-cell studies. Specifically, both conditions resulted in libraries with comparable complexity as determined by the linear relationship between the number of sequencing reads and detected transcripts or genes (Fig. 6a, b). We were able to detect similar numbers of genes by cumulating information over single cells (Fig. 6c). Average gene expression levels were highly correlated (Fig. 6d). Due to the introduced batch effects, we could detect patterns in the transcriptional profile of the most variable genes (Fig. 6e); however, these did not bias the annotation to cell subpopulation after hierarchical clustering and t-SNE representation (Fig. 6f, g). We were able to identify transit amplifying (TA) cells, secretory enteroendocrine cells, and differentiated enterocytes in both conditions (Fig. 6g, h); the major cell types present in the colon mucosa. The single-cell transcriptome data enabled us to assign colon cell types to cell clusters using marker genes [9], such as *Reg4* (secretory cells), *Apoa1* (enterocytes), or ribosomal proteins (TA cells) (Fig. 6h). Importantly, all cell types were identified in equal proportions by fresh and cryopreserved cells, excluding a systematic bias introduced by the conservation process (χ^2^ test, *p* = 0.95; Fig. 6g, h). We conclude that the conservation process did not alter the transcriptional profile of single colon mucosa cells and that both, single-cell sequencing of fresh and conserved tissues, is equally suitable to extract biologically relevant information, such as cell-type-specific programs.

**Fig. 7:**
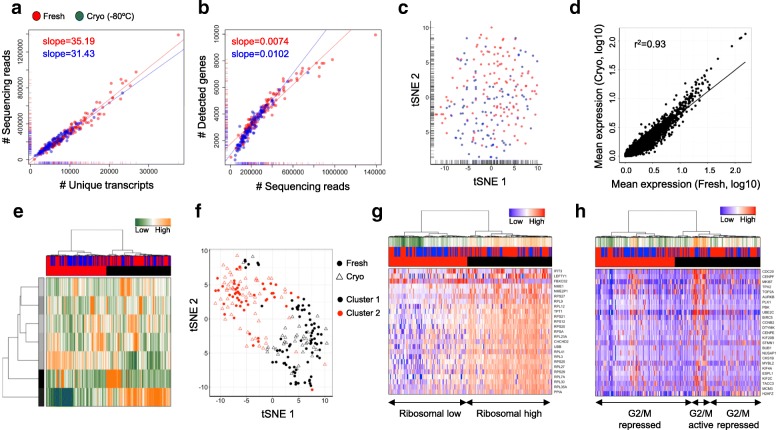
Comparative analyses of single-cell transcriptome data from fresh (*red*) and cryopreserved (*blue*) patient-derived orthotopic ovarian tumor xenograft cells. **a**, **b** Comparative analysis of the number of sequencing reads and detected transcripts (**a**) or genes (**b**) using a linear model. The slope of the regression line was calculated separately for fresh and cryopreserved cells. **c** Gene expression variances displayed as t-SNE representation using the 100 most variable genes. **d** Linear regression model comparing average gene expression levels of expressed genes. The coefficient of determination (r^2^) is indicated. **e** Hierarchical clustering of single cells based on transcriptional programs (defined by Gene Ontology) and correlating gene sets [21]. Transcriptional programs and gene clusters are summarized in aspects. Displayed are the most variable aspects (*rows*) and their importance (*row colors*). Cells are assigned to condition (fresh: *red*; cryopreserved: *blue*) and clusters. **f** A t-SNE representation of similarities between cells using distances and cluster identities (as in **e**). Conditions are indicated (fresh: *circle*; cryopreserved: *triangle*). **g**, **h** Hierarchical clustering of single cells (as in **e**). Displayed are the expression levels of the 25 most variable ribosomal genes (**g**) and genes implicated in cell cycle (G2/M checkpoint, **h**). Gene set expression levels are summarized (first panel: high: *orange*; low: *green*) and cells are assigned to condition (second panel: fresh: *red*; cryopreserved: *blue*) and clusters

Finally, we applied our method to a PDOX in mouse. It is of note that digestion of the tumor sample to single-cell solution prior to cryopreservation resulted in an increased proportion of damaged cells (56% damaged cells), compared to the sample conserved as minced material (26% damaged cells). The cryopreserved ovarian clear cell carcinoma orthoxenograft (passage #2) was processed simultaneously with a matched freshly resected PDOX (passage #3). Therefore, the tumor was cryopreserved for three months and simultaneously sub-cultured in a mouse to obtain a fresh matched specimen. Consistent with prior observations, single transcriptome profiles of fresh or conserved tumor cells did not differ in their library complexity (Fig. 7a, b), transcriptional profiles (Fig. 7c), or gene expression levels (Fig. 7d). No significantly differentially expressed genes could be detected (Additional file 2: Table S9). Single cells from both conditions were able to detect a large tumor cell subpopulations (Fig. 7e, f) with an elevated expression level of ribosomal protein-coding genes (Fig. 7g) and a minor population with an activated G2/M checkpoint profile (Fig. 7h), further highlighting tissue conservation to be possible for various experimental designs, including tumor samples. We observed a proportional bias between the conditions within the putative tumor subpopulations when clustering cells using variable gene sets (χ^2^ test, *p* = 0.0002, Fig. 7g). Although we cannot finally exclude the preservation having caused the proportional shift, variant clonal heterogeneity presents a highly frequent feature of serial passaging of PDOX samples [13]. Interestingly, these differences were absent when analyzing the most variable genes separately (χ^2^ test, *p* = 0.87, Additional file 1: Figure S16), suggesting that gene set-based hierarchical clustering might be too restrictive to sensitively assign subclonal structures in heterogeneous cancer samples.

## Conclusions

Using the here-established cryopreservation method, single-cell transcriptome profiles from cells and tissues did not differ from freshly processed material. The method constitutes a straightforward and powerful tool to broaden the scope of single-cell genomics study designs. Importantly, cryopreservation can be readily implemented into standard single-cell genomics workflows, without modifications of established protocols. Following the evaluation of the applicability in 3’-tag (MARS-Seq) and full-length (Smart-seq2) transcriptome sequencing techniques, other single-cell RNA-seq methods are likely to result in similar outcomes [2, 8, 14]. Although recent work described the value of nuclear RNA analysis [4, 15], the content from viable cells results in more complex transcriptomes, allowing accurate cell phenotyping. It is of note that a certain degree of cell damage introduced by the cryopreservation procedure has to be taken into account when working with low-input material. Furthermore, different downstream applications, including genome or epigenome sequencing, might also benefit from this method. Cryopreservation was previously described to conserve open chromatin structures in ATAC sequencing experiments [16], pointing to a wide application spectrum of cryopreserved material.

In conclusion, the conservation process we present here does not modify transcriptional profiles of single cells taken from cell culture or tissues. Cells cryopreserved by our method are equally well suited as fresh cells to extract relevant biological information, such as cell-type-specific programs. This substantially broadens the scope of applications in single-cell transcriptomics and could constitute a paradigm shift for single-cell study designs.

## Methods

### Cell line sample preparation

Human cell lines HEK293 (human embryonic kidney cells) and K562 (human leukemia cells) were acquired from the German Collection of Microorganisms and Cell Cultures (DSMZ). NIH3T3 (mouse embryo fibroblasts) and MDCK (canine adult kidney cells) were kindly provided by Dr. Manel Esteller (IDIBELL, Spain). HEK293, NIH3T3, and MDCK were maintained in DMEM (10% fetal bovine serum (FBS); 1% Penicillin/Streptomycin) at 37 °C (5% CO_2_). K562 suspension cells were cultured in RPMI (10% FBS; 1% Penicillin/Streptomycin) at 37 °C (5% CO_2_). For cryopreservation, cells were trypsinized, pelleted, and resuspended in freezing solution (10% DMSO; 10% heat-inactivated FBS; 80% DMEM). Subsequently, cells were frozen with gradually decreasing temperatures (1 °C/min) to –80 °C (cryopreserved). Cryopreserved cells lines were stored for one week at –80 °C or liquid nitrogen before further processing. For single-cell analysis, cryopreserved cells were rapidly thawed in a water bath with continuous agitation and placed into 25 mL of cold 1× HBSS. Fresh cells were trypsinized, pelleted, and resuspended in 1× HBSS. Before sorting, cells from both conditions were filtered (70 μm nylon mesh) and propidium iodide staining identified dead/damaged cells. Cells were FACS sorted into MARS-Seq or Smart-seq2 plates using FACS Aria Fusion (Becton Dickinson). To avoid batch effects, fresh and cryopreserved single cells were sorted into the same plates and distributed over both sequencing pools.

### Peripheral blood sample preparation

Whole blood was collected from a healthy donor into EDTA tubes (Becton, Dickinson & Co). Separation of PBMC was performed with Ficoll-Paque PREMIUM (GE Healthcare) according to the manufacturer’s instructions. Briefly, for density gradient separation, 4 mL of blood was added to 3 mL of Ficoll-Paque PREMIUM and centrifuged at 400 × g for 30 min at 18 °C without brake. PBMC layer was recuperated and washed twice with PBS at 400 × g for 15 min at 18 °C. PBMC were resuspended in freezing solution (10% DMSO, 90% non-inactivated FBS) and frozen by gradually decreasing temperature (1 °C/min) to –80 °C (cryopreserved). After storage for one week at –80 °C, the cryopreserved sample was rapidly thawed in a water bath in continuous agitation and placed into 25 mL of cold 1× HBSS. Cells were washed once in ice-cold 1× HBSS and resuspended in DMEM before sorting. To avoid batch effects, freshly isolated PBMC were sorted in parallel with the cryopreserved material and distributed over the same sequencing pools. Therefore, blood from the same donor was isolated as described above and directly resuspended in DMEM. Dead and damaged cells were identified by propidium iodide staining.

### Primary colon sample preparation

Female athymic nu/nu mice (Harlan) aged four to six weeks were housed in individually ventilated cages on a 12-h light-dark cycle at 21–23 °C and 40–60% humidity. Mice were allowed free access to an irradiated diet and sterilized water. Primary mouse colon was dissected from an athymic nu/nu mouse and placed on ice. The sample was divided and half of the colon was immediately prepared for single-cell separation, while the other half was minced on ice, placed into freezing solution (10% DMSO, 90% non-inactivated FBS) and frozen by gradually decreasing temperature (1 °C/min) to –80 °C (cryopreserved). After storage for one week at –80 °C, the sample was rapidly thawed in a water bath in continuous agitation and placed into 25 mL of cold 1× HBSS. For single-cell separation the fresh and conserved samples were minced on ice and enzymatically digested in 5 mL 1× HBSS and 83 μL collagenase IV (10,000 U/mL) for 10 min at 37 °C. Single cells were separated by passing the sample through a 0.9-mm needle and filtration (70 μm nylon mesh). Cells were washed once in ice-cold 1× HBSS and resuspended in DMEM before sorting. Dead and damaged cells were identified by propidium iodide staining. For practical reasons (tissue derived from one single mouse), fresh and cryopreserved single cells could not be sorted into the same plate.

### Orthotopic tumor engraftment

To analyze matched fresh and cryopreserved viable tumor samples, we generated an ovarian and lung orthotopic tumor model, referred to as Orthoxenograft® or PDOX. Therefore, we implanted a primary clear cell ovarian carcinoma and a lung adenocarcinoma metastasis into the ovaries and brain of athymic nu/nu mice (matched organ of origin), respectively. Briefly, the primary tumor specimens were obtained at the University Hospital of Bellvitge or the Vall d’Hebron University Hospital (Barcelona, Spain). The selected ovarian carcinoma patient had not received cisplatin-based chemotherapy. Non-necrotic tissue pieces (~2–3 mm^3^) from a resected clear cell ovarian carcinoma and a lung adenocarcinoma metastasis were selected and placed into DMEM, supplemented with 10% FBS and 1% penicillin/streptomycin at room temperature. Under isofluorane-induced anesthesia, animals were subjected to a lateral laparotomy, their ovaries or brain exposed, and tumor pieces anchored to the ovary surface with prolene 7.0 sutures [17, 18]. Tumor growth was monitored two to three times per week. When the tumor grew, it was harvested and cut into small fragments. Subsequently, it was transplanted into a new animal or cryopreserved at –80 °C as a viable tumor (as described above). After 107 days, the ovarian tumor was newly resected from the mouse and processed together with the matched cryopreserved sample (maintained at –80 °C) for single-cell separation and sorting. The lung adenocarcinoma metastasis was cryopreserved for 192 days before further processing. The morphology of the primary tumor and the engrafted tumor was compared by H&E staining in paraffin-embedded sections. For cell separation, the cryopreserved sample was rapidly thawed in a water bath in continuous agitation and placed into 25 mL of cold 1× HBSS. For single-cell isolation, the fresh and conserved samples were enzymatically digested in 5 mL 1× HBSS and 83 μL collagenase IV (10,000 U/mL) for 15 min at 37 °C. Single cells were separated by passing the sample through a 0.9-mm needle and filtration (70 μm nylon mesh). Cells were washed once in ice-cold 1× HBSS and resuspended in DMEM before sorting. In order to enrich human cells during the sorting procedure, tumor cells were stained for 1 h at 4 °C with α-EpCam (CD326, eBioscience, 1:100). Propidium iodide staining identified dead/damaged cells. To avoid batch effects, fresh and cryopreserved single cells were sorted into the same plates and distributed over both sequencing pools.

### Library preparation and sequencing

To construct single-cell libraries from polyA-tailed RNA, we applied MARS-Seq [5, 6]. Briefly, single cells were FACS-sorted into 384-well plates, containing lysis buffer (0.2% Triton (Sigma-Aldrich); RNase inhibitor (Invitrogen)) and reverse-transcription (RT) primers. The RT primers contained the single-cell barcodes and unique molecular identifiers (UMIs) for subsequent de-multiplexing and correction for amplification biases, respectively. Single-cell lysates were denatured and immediately placed on ice. The RT reaction mix, containing SuperScript III reverse transcriptase (Invitrogen), was added to each sample. In the RT reaction, spike-in artificial transcripts (ERCC, Ambion) were included at a dilution of 1:16 × 10^6^ per cell. After RT, the complementary DNA (cDNA) was pooled using an automated pipeline (epMotion, Eppendorf). Unbound primers were eliminated by incubating the cDNA with exonuclease I (NEB). A second pooling was performed through cleanup with SPRI magnetic beads (Beckman Coulter). Subsequently, pooled cDNAs were converted into double-stranded DNA with the Second Strand Synthesis enzyme (NEB), followed by clean up and linear amplification by T7 in vitro transcription overnight. Afterwards, the DNA template was removed by Turbo DNase I (Ambion) and the RNA was purified with SPRI beads. Amplified RNA was chemically fragmented with Zn2+ (Ambion), then purified with SPRI beads. The fragmented RNA was ligated with ligation primers containing a pool barcode and partial Illumina Read1 sequencing adapter using T4 RNA ligase I (NEB). Ligated products were reversed transcribed using the Affinity Script RT enzyme (Agilent Technologies) and a primer complementary to the ligated adapter, partial Read1. The cDNA was purified with SPRI beads. Libraries were completed through a polymerase chain reaction (PCR) step using the KAPA Hifi Hotstart ReadyMix (Kapa Biosystems) and a forward primer that contains Illumina P5-Read1 sequence and the reverse primer containing the P7-Read2 sequence. The final library was purified with SPRI beads to remove excess primers. Library concentration and molecular size were determined with High Sensitivity DNA Chip (Agilent Technologies). The libraries consisted of 192 single-cell pools. Multiplexed pools (2) were run in one Illumina HiSeq 2500 Rapid two lane flow cell following the manufacturer’s protocol. Primary data analysis was carried out with the standard Illumina pipeline. We produced 52 nt of transcript sequence reads for the cell lines, the PBMC, and the mouse colon tissue and 83 nt for the tumor xenograft sample.

Full-length single-cell RNA-seq libraries were prepared using the Smart-seq2 protocol [12] with minor modifications. Briefly, freshly harvested or cryopreserved (1 week at –80 °C) single cells were sorted into 96-well plates containing the lysis buffer. Reverse transcription was performed using SuperScrpit II (Invitrogen) in the presence of oligo-dT30VN, template-switching oligonucleotides and betaine. The cDNA was amplified using the KAPA Hifi Hotstart ReadyMix (Kappa Biosystems), ISPCR primer, and 20 cycles of amplification. Following purification with Agencourt Ampure XP beads (Beckmann Coulter), product size distribution and quantity were assessed on a Bioanalyzer using a High Sensitvity DNA Kit (Agilent Technologies). A total of 200 ng of the amplified cDNA was fragmented using Nextera® XT (Illumina) and amplified with indexed Nextera® PCR primers. Products were purified twice with Agencourt Ampure XP beads and quantified again using a Bioanalyzer High Sensitvity DNA Kit. Sequencing of Nextera® libraries from 95 cells was carried out using two sequencing lanes on a HSeq2000 (Illumina).

### Data processing

The MARS-Seq technique takes advantage of two-level indexing that allows the multiplexed sequencing of 192 cells per pool and multiple pools per sequencing lane. Sequencing was carried out as paired-end reads, wherein the first read contains the transcript sequence and the second read the cell barcode and UMIs. Quality check of the generated reads was performed with the FastQC quality control suite. Samples that reached the quality standards were then processed to deconvolute the reads to single-cell level by de-multiplexing according to the cell and pool barcodes. Reads were filtered to remove polyT sequences. Sequencing reads from human, mouse, or canine cells were mapped with the RNA pipeline of the GEMTools 1.7.0 suite [19] using default parameters (6% of mismatches, minimum of 80% matched bases, and minimum quality threshold of 26) and the genome references for human (Gencode release 24, assembly GRCh38.p5), mouse (Gencode release M8, assembly GRCm38.p4), and dog (Ensembl v84, assembly CanFam3.1). The analysis of spike-in control RNA content allowed us to identify empty wells and barcodes with more than 15% of reads mapping to spike-in artificial transcripts were discarded. In addition, cells with less than 60% of reads mapping on the reference genome or more than 2 × 10^6^ total reads were discarded. Gene quantification was performed using UMI corrected transcript information to correct for amplification biases, collapsing read counts for reads mapping on a gene with the same UMI (allowing an edit distance up to two nucleotides in UMI comparisons). Only unambiguously mapped reads were considered. Genes not expressed in at least 5% of the cells were discarded. Thresholds were set to reduce technical noise, but to conserve the sensitivity to identify low frequency outlier cell populations and to capture differences between fresh and cryopreserved cells.

### Data analysis

To estimate systematic biases introduced by the conservation technique, single cells from both conditions were compared using commonly used data pre-processing strategies and different metrics to assess similarities between cells. Statistical analyses shown in this manuscript were carried out using R, version 3.3.0. Functions referred to below belong to the R *stats* package when not indicated otherwise.

#### Heterogeneity analysis

Fresh and cryopreserved datasets were independently filtered for low-quality cells, removing cells with a relatively low number of detected genes (Additional file 2: Table S1). The absolute threshold was variable and depended on the experiment and sequencing protocol (Additional file 1: Figure S1b, e). The thresholds were set based on the distribution of the number of non-zero count genes per cell (minimum number of genes detected), removing cells having more than 2 median absolute deviations (MAD) below the median of the minimum number of genes. In addition to filter for genes detected in > 5% of cells, genes in the lower quartile of average gene expressions were discarded.

Count data from fresh and cryopreserved cells was initially analyzed separately and then genes of both datasets were merged resulting in a joint gene-cell matrix for each experiment. To detect genes differentially expressed and to perform heterogeneity analysis, gene expression levels were modeled as a mixture of negative binomial and Poisson distributions, using *scde* package [20]. This method allows gene expression inferences from amplified and drop-out events. To fit cell models, modeling parameters were adapted to the dataset size and to the use of UMI (MARS-Seq) or read (Smart-seq2) counts. The quality of models was evaluated with the correlation value to the expected magnitude, which was positive for all cells. Further, the probability distribution of drop-out events for each sample appeared highly and negatively correlated to the expression magnitude, showing the value 1 associated to zero magnitudes. Gene expression variances were normalized to the expected variance based on the models to determine the MVG. Variability introduced during the experimental phase due to the library preparation in distinct pools has been taken into account, normalizing for technical aspects during PAGODA [21] data processing. PCA and t-SNE representation were performed using the top 100 genes from the MVG list (Figs. 1e, f, 3c, d, 4c, d, 5e, 6e, and 7c; Additional file 1: Figures S6–9, S12). Both methods classify in an unsupervised manner by grouping most similar cells into clusters, while the t-SNE algorithm also captures non-linear relationships. Further, MVG were calculated separately for fresh and cryopreserved HEK293, NIH3T3, and MDCK cells to assess the overlap of genes. To be able to compare this value, we randomly subsampled only fresh cells using 100 permutations and determined the distribution of overlapping genes. Random resampling has been performed without replacement (using the *sample* function), dividing each fresh sample into two complementary sets of cells. For each paired group, we computed the number of overlapping genes and evaluated their distribution and average overlap. The same strategy was repeated comparing the fresh and cryopreserved groups sampled with equal cell numbers as the only-fresh groups.

#### Subpopulation analysis

We looked at the variance explained by the first principal component of Gene Ontology, de novo, or custom gene sets to define clusters of gene sets (aspects) using PAGODA [21]. The same package allows the identification of principal aspects of heterogeneity, identifying the most overdispersed gene sets. In order to reduce redundancy, gene sets showing correlating expression patterns were integrated into aspects using a distance threshold of 0.9. Subsequently, cells were clustered based on a weighted correlation of genes that drive the aspects and the heatmaps highlight the most variable aspects (Figs. 2g, 3g, 4g, 5f, 6f, and 7e; Additional file 1: Figure S11a, d). Further, correlation from the hierarchical clustering were used to visualize cells in two dimensions through a t-SNE plot, allowing to define clusters (Figs. 2h, 3h, 4h, 5g, 6g, and 7f; Additional file 1: Figure S11b, e). Cell states or types following Pagoda cluster identification were assigned using the most variable genes (Figs. 2i, 3i, 4i, 5h, 6h, and 7g, h). Pagoda defines these genes using a weighted PCA to take into account drop-out events and other technical bias. The displayed genes represent signatures (e.g. G2/M checkpoint) or variable genes in de novo assigned gene sets. To cluster blood cell subpopulations and to assign phenotypes, we integrated cell-type-specific sets derived from the GSEA database [22], Björklund et al. [23] and Palmer et al. [24]. Mouse colon cell types were identified using marker genes defined in Grün et al. [9]. Cell subpopulations in the ovarian tumor xenograft were characterized using Gene Ontology enrichment analysis and G2/M checkpoint genes. Apoptosis (Hallmark_Apoptosis; M5902) and G2/M (Hallmark_G2/M_CHECKPOINT; M5901) gene sets were derived from the GSEA database [22].

#### Differential gene expression analysis

We compared transcriptional profiles between fresh and cryopreserved cells using *scde* [20]. Most datasets revealed that the relative contribution of each gene between the two groups of cells was highly comparable (Additional file 2: Tables S2–S9). A low number of genes were identified to be differentially expressed in the Smart-seq2 datasets (Additional file 1: Figures S13c, S14c). Here, the sample size was very small (n = 24) and the variance could be explained by sampling bias.

#### Expression correlation analysis

Differences between gene expression profiles were investigated by correlating relative and absolute gene counts of the entire gene set (Figs. 2d–f, 5d, 6d, and 7d; Additional file 1: Figure S10i–k). Linear regression models pointed to a strong linear correlation (r^2^ ~ 0.9) between the means of the two groups that were computed considering log-average gene counts. For cell-wise comparisons (Figs. 2a, c, 3e, and 4e; Additional file 1: Figure S10a–d), gene expression levels of the 500 most expressed genes were scaled based on UMI counts to correct for differences in library sizes between cells and normalized by quantile normalization with the *qnorm* function. Pearson’s correlation matrices were calculated for 202 randomly selected cells per experiment/condition with the *cor* function and represented using the *corrplot* library.

#### Cumulative gene counts

The cumulative number of genes detected over multiple cells was assessed by calculating the mean of total genes retrieved after 100 permutations of an increasing number of randomly sampled cells (*sample* function). Cells in the lower quartile of library sizes were discarded and the remaining cells were downsampled to the lowest library size with the *downsample.counts* function from the *metaseqR* package. Results are represented as cumulative gene counts (Figs. 1b, 3b, 4b, 5c, and 6c; Additional file 1: Figure S2a–d).

## Additional files

Additional file 1: Figures S1–16.
**Figure S1.** Single cell transcriptome sequencing of 670 fresh and 816 cryopreserved cells using the MARS-Seq (**a**-**c**) and the Smart-seq2 (**d**, **e**) library preparation protocols. Samples included two human (HEK293 and K562), one mouse (NIH3T3) and one canine (MDCK) cell line, peripheral blood mononuclear cells (PBMC), a primary mouse colon sample and orthotopic tumor xenografts (patient-derived orthotopic xenograft, PDOX). Analyses were split by replicate experiments and conditions. Displayed are the total number of reads per cell (**a**,**d**), the total number of detected genes per cell (**b**,**e**) and the number of detected transcripts (UMI counts) per cell (**c**). The results are displayed as boxplot indicating median values (black bar) per experiment and condition. **Figure S2.** (**a**-**d**) Cumulative gene counts split by fresh (**red**) or cryopreserved (**blue**) cells and analyzed using randomly sampled cells (average of 100 permutations). Displayed are results for K562 (experiment 1, **a**), HEK293 (experiment 1, **b**), NIH3T3 (**c**) and MDCK (**d**) cell lines. **Figure S3.** Distribution of sequencing reads numbers per single cell split by conditions (fresh (**red**); cryopreserved -80ºC: **blue**; cryopreserved liquid nitrogen: **green**). Displayed are the distributions for all MARS-Seq experiments (**a**-**h**) indicating the median number of reads per single cell (horizontal lines). Experiment types are indicated. **Figure S4.** MARS-Seq library complexity assessment of fresh (**red**) or cryopreserved (**blue**) cells using the number of uniquely detected transcripts. (**a**-**d**) Comparative analysis of the number of sequencing reads and detected transcripts using a linear model. The slope of the regression line was calculated separately for fresh and cryopreserved cells. Displayed are results for K562 (experiment 1, **a**), HEK293 (experiment 1, **b**), NIH3T3 (**c**) and MDCK (**d**) cell lines. **Figure S5.** MARS-Seq library complexity assessment of fresh (**red**) or cryopreserved (**blue**) cells using the total number of detected genes. (**a**-**d**) Comparative analysis of the number of sequencing reads and detected transcripts using a linear model. The slope of the regression line was calculated separately for fresh and cryopreserved cells. Displayed are results for K562 (experiment 1, **a**), HEK293 (experiment 1, **b**), NIH3T3 (**c**) and MDCK (**d**) cell lines. **Figure S6.** Comparative analyses of single cell transcriptome variance from fresh (**red**) and cryopreserved (**blue**) cell lines. Gene expression variances between cells are displayed as principal component analysis (PCA, upper panel **a**-**d**) or t-distributed stochastic neighbor embedding (t-SNE, lower panel **e**-**h**) using the 100 most variable genes. The displayed experiments include K562 (experiment 1; **a**,**e**), HEK293 (experiment 1; **b**,**f**), NIH3T3 (**c**,**g**) and MDCK (**d**,**h**). **Figure S7.** Joint analyses of single cells from fresh (**red**), cryopreserved at -80ºC (**blue**) and cryopreserved in liquid nitrogen (**green**) HEK293 cells from experiment 1 (circles) and 2 (triangles). Gene expression variances between cells are displayed as principal component analysis (PCA, **a**) or t-distributed stochastic neighbor embedding (t-SNE, **b**) using the 100 most variable genes. **Figure S8.** Comparative analyses of single cells from fresh (**red**) and cryopreserved (**blue**) cell lines. Gene expression variances between cells are displayed as t-distributed stochastic neighbor embedding (t-SNE) using the 100 most variable genes and the indicated perplexity parameter values. The displayed experiments include K562 (experiment 1), HEK293 (experiment 1 and 2), NIH3T3 and MDCK. **Figure S10.** Correlation analysis of gene expression levels split by conditions (fresh (**red**); cryopreserved -80ºC: **blue**; cryopreserved liquid nitrogen: **green**). (**a**-**d**) Pearson’s correlation analysis between 20 randomly selected fresh and cryopreserved cells displaying the correlation coefficient (r2). (**e**-**h**) Distribution of Pearson’s correlation coefficients (r2) within and between processing conditions. The median coefficients are indicated. (**i**-**k**) Linear regression model comparing average gene expression levels of expressed genes. The coefficient of determination (r2) is displayed. Cell lines and experiments are indicated within the figures. **Figure S11.** Cell subtype analysis of HEK293 (experiment 1) cells split by fresh (**a**-**c**) and cryopreserved (**d**-**f**) cells. (**a**,**d**) Hierarchical clustering of single cells based on transcriptional programs (defined by gene ontology) and correlating genes [21]. Transcriptional programs and gene clusters are summarized in aspects (orange: overrepresented; green: underrepresented). Displayed are the most variable aspects (rows) and their importance (row colors). Cells are assigned to condition (fresh: red; cryopreserved: blue) and clusters. (**b**, **e**) A t-distributed stochastic neighbor embedding (t-SNE) representation of similarities between cells using previous defined distances and cluster identity (as in **a**, **d**). (**c**, **f**) Hierarchical cluster of single cells (as in **a**,**d**) displaying the 25 most variable cell cycle genes (G2/M checkpoint). Expression levels of the cell cycle signature are summarized (1st panel; orange: high, green: low) and clusters are indicated. **Figure S12.** Analyses of Smart-seq2-derived single cell transcriptomes from a cryopreserved patient-derived xenograft (PDOX) tumor. (**a**) Displayed are the sequencing read distribution following RNA library preparation of full-length transcripts. Each line represents a single cell and transcript sizes are scaled from (0-100). (**b**,**c**) Comparative analyses of single cells from fresh (**circles**) and cryopreserved (**triangles**) samples. The displayed experiments include single cells from HEK293 cells (blue), K562 cells (orange) and the PDOX (green) sample. Gene expression variances between cells are displayed as principal component analysis (PCA, **b**) and t-distributed stochastic neighbor embedding (t-SNE, **c**) using the 100 most variable genes. **Figure S13.** Cell subtype analysis of HEK293 cells analyzed by Smart-seq2 split by fresh (**a**) and 5 cryopreserved (**b**) cells. (**a**,**b**) Hierarchical clustering of single cells based on transcriptional programs (defined by gene ontology) and correlating genes [21] displaying the 25 most variable cell cycle genes (G2/M checkpoint). Expression levels of the cell cycle signature are summarized (1st panel; orange: high, green: low) and clusters are indicated. (**c**) Significantly differentially expressed genes between fresh and cryopreserved HEK293 cells (p < 0.01). **Figure S14.** Cell subtype analysis of K562 cells analyzed by Smart-seq2 split by fresh (**a**) and cryopreserved (**b**) cells. (**a**,**b**) Hierarchical clustering of single cells based on transcriptional programs (defined by gene ontology) and correlating genes [21] displaying the 25 most variable cell cycle genes (G2/M checkpoint). Expression levels of the cell cycle signature are summarized (1st panel; orange: high, green: low) and clusters are indicated. (**c**) Significantly differentially expressed genes between fresh and cryopreserved K562 cells (p < 0.01). **Figure S15.** Cell subtype analysis of fresh (**red**) and cryopreserved (**blue**) peripheral blood mononuclear cells analyzed by MARS-Seq. (**a**-**d** ) Hierarchical clustering of single cells based on transcriptional programs (defined by gene ontology) and correlating genes [21] (as defined in Fig. 5f) displaying the 25 most variable genes within the signatures for cytotoxic T-cells (a), memory T-cells (**b**), B-cells (**c**) and myeloid cells (**d**). Expression levels of all signature genes are summarized (1st panel; orange: high, green: low) and conditions (2nd panel: fresh: red; cryopreserved: blue) and clusters are indicated. The lower plots summarize the signature expression levels for each cell (dots) and cluster (box). Inferred cell types are indicated (BC: Bcells, CytoTC: cytotoxic T-cells, MemTC: memory T-cells, Myd: myeloid cells). **Figure S16.** Hierarchical clustering of single cells from an ovarian tumor PDOX based on the most variable genes. Cells are assigned to conditions (2nd panel; fresh: red; cryopreserved: blue) and clusters (3rd panel). Displayed are the 25 most variable genes implicated in cluster formation. Expression levels of the genes are summarized (1st panel; orange: high, green: low). (PDF 6319 kb)
Additional file 2: Tables S1–9.
**Table S1.** Overview table of experiments. **Table S2.** Differential gene expression between fresh and cryopreserved K562 cells. (MARS-Seq; top 40 genes). **Table S3.** Differential gene expression between fresh and cryopreserved HEK293 cells. (MARS-Seq; top 40 genes). **Table S4.** Differential gene expression between fresh and cryopreserved NIH3T3 cells. (MARS-Seq; top 40 genes). **Table S5.** Differential gene expression between fresh and cryopreserved MDCK cells. (MARS-Seq; top 40 genes). **Table 6**: Differential gene expression between fresh and cryopreserved HEK293 cells. (SMARTseq2; top 40 genes). **Table 7**: Differential gene expression between fresh and cryopreserved K562 cells. (SMARTseq2; top 40 genes). **Table S8**: Differential gene expression between fresh and cryopreserved PBMC. (MARS-Seq; top 40 genes). **Table S9**: Differential gene expression between a fresh and cryopreserved PDOX. (MARSseq; top 40 genes). (PDF 40 kb)

## Abbreviations

DMSO: Dimethyl-sulfoxide
ERCC: External RNA control consortium
FACS: Fluorescence-activated cell sorting
FBS: Fetal bovine serum
GSEA: Gene set enrichment analysis
MARS-Seq: Massively parallel single-cell RNA sequencing
PAGODA: Pathway and gene set overdispersion analysis
PCA: Principal component analyses
PDOX: Patient-derived orthotopic xenograft
t-SNE: T-distributed stochastic neighbor embedding
UMI: Unique molecular identifier
