# Supplementary material for: Single-cell transcriptome conservation in cryopreserved cells and tissues
Source: Genome Biol. 2017 Mar 1;18:45. doi: 10.1186/s13059-017-1171-9 (PMC5333448; doi:10.1186/s13059-017-1171-9)
Supplement: Additional file 1: Figures S1–16. — Figure S1. Single cell transcriptome sequencing of 670 fresh and 816 cryopreserved cells using the MARS-Seq (a-c) and the Smart-seq2 (d, e) library preparation protocols. Samples included two human (HEK293 and K562), one mouse (NIH3T3) and one canine (MDCK) cell line, peripheral blood mononuclear cells (PBMC), a primary mouse colon sample and orthotopic tumor xenografts (patient-derived orthotopic xenograft, PDOX). Analyses were split by replicate experiments and conditions. Displayed are the total number of reads per cell (a,d), the total number of detected genes per cell (b,e) and the number of detected transcripts (UMI counts) per cell (c). The results are displayed as boxplot indicating median values (black bar) per experiment and condition. Figure S2. (a-d) Cumulative gene counts split by fresh (red) or cryopreserved (blue) cells and analyzed using randomly sampled cells (average of 100 permutations). Displayed are results for K562 (experiment 1, a), HEK293 (experiment 1, b), NIH3T3 (c) and MDCK (d) cell lines. Figure S3. Distribution of sequencing reads numbers per single cell split by conditions (fresh (red); cryopreserved -80ºC: blue; cryopreserved liquid nitrogen: green). Displayed are the distributions for all MARS-Seq experiments (a-h) indicating the median number of reads per single cell (horizontal lines). Experiment types are indicated. Figure S4. MARS-Seq library complexity assessment of fresh (red) or cryopreserved (blue) cells using the number of uniquely detected transcripts. (a-d) Comparative analysis of the number of sequencing reads and detected transcripts using a linear model. The slope of the regression line was calculated separately for fresh and cryopreserved cells. Displayed are results for K562 (experiment 1, a), HEK293 (experiment 1, b), NIH3T3 (c) and MDCK (d) cell lines. Figure S5. MARS-Seq library complexity assessment of fresh (red) or cryopreserved (blue) cells using the total number of detected genes. (a-d) Comparative analysis of [file 13059_2017_1171_MOESM1_ESM.pdf]

Additional file 1:

Supplementary figure legends 1-16

Supplementary figures 1-16

Supplementary figure legends

Figure S1 Single cell transcriptome sequencing of 670 fresh and 816 cryopreserved cells using the MARS-Seq (**a-c**) and the Smart-seq2 (**d,e**) library preparation protocols. Samples included two human (HEK293 and K562), one mouse (NIH3T3) and one canine (MDCK) cell line, peripheral blood mononuclear cells (PBMC), a primary mouse colon sample and orthotopic tumor xenografts (patient-derived orthotopic xenograft, PDOX). Analyses were split by replicate experiments and conditions. Displayed are the total number of reads per cell (**a,d**), the total number of detected genes per cell (**b,e**) and the number of detected transcripts (UMI counts) per cell (**c**). The results are displayed as boxplot indicating median values (black bar) per experiment and condition.

Figure S2 (**a-d**) Cumulative gene counts split by fresh (**red**) or cryopreserved (**blue**) cells and analyzed using randomly sampled cells (average of 100 permutations). Displayed are results for K562 (experiment 1, **a**), HEK293 (experiment 1, **b**), NIH3T3 (**c**) and MDCK (**d**) cell lines.

Figure S3 Distribution of sequencing reads numbers per single cell split by conditions (fresh (**red**); cryopreserved -80ºC: **blue**; cryopreserved liquid nitrogen: **green**). Displayed are the distributions for all MARS-Seq experiments (**a-h**) indicating the median number of reads per single cell (horizontal lines). Experiment types are indicated.

Figure S4 MARS-Seq library complexity assessment of fresh (**red**) or cryopreserved (**blue**) cells using the number of uniquely detected transcripts. (**a-d**) Comparative analysis of the number of sequencing reads and detected transcripts using a linear model. The slope of the regression line was calculated separately for fresh and cryopreserved cells. Displayed are results for K562 (experiment 1, **a**), HEK293 (experiment 1, **b**), NIH3T3 (**c**) and MDCK (**d**) cell lines.

Figure S5 MARS-Seq library complexity assessment of fresh (**red**) or cryopreserved (**blue**) cells using the total number of detected genes. (**a-d**) Comparative analysis of the number of sequencing

reads and detected transcripts using a linear model. The slope of the regression line was calculated separately for fresh and cryopreserved cells. Displayed are results for K562 (experiment 1, **a**), HEK293 (experiment 1, **b**), NIH3T3 (**c**) and MDCK (**d**) cell lines.

Figure S6 Comparative analyses of single cell transcriptome variance from fresh (**red**) and cryopreserved (**blue**) cell lines. Gene expression variances between cells are displayed as principal component analysis (PCA, upper panel **a-d**) or t-distributed stochastic neighbor embedding (t-SNE, lower panel **e-h**) using the 100 most variable genes. The displayed experiments include K562 (experiment 1; **a,e**), HEK293 (experiment 1; **b,f**), NIH3T3 (**c,g**) and MDCK (**d,h**).

Figure S7 Joint analyses of single cells from fresh (**red**), cryopreserved at -80ºC (**blue**) and cryopreserved in liquid nitrogen (**green**) HEK293 cells from experiment 1 (circles) and 2 (triangles). Gene expression variances between cells are displayed as principal component analysis (PCA, **a**) or t-distributed stochastic neighbor embedding (t-SNE, **b**) using the 100 most variable genes.

Figure S8 Comparative analyses of single cells from fresh (**red**) and cryopreserved (**blue**) cell lines. Gene expression variances between cells are displayed as t-distributed stochastic neighbor embedding (t-SNE) using the 100 most variable genes and the indicated perplexity parameter values. The displayed experiments include K562 (experiment 1), HEK293 (experiment 1 and 2), NIH3T3 and MDCK.

Figure S9 Single cell transcriptome variance across conditions and tissue types. Gene expression variances between HEK293 (experiment 2) and K562 (experiment 1) cells are displayed as principal component analysis (PCA, **a**) or t-distributed stochastic neighbor embedding (t-SNE, **b**) using the 150 most variable genes. Cell line identity and processing conditions are indicated.

Figure S10 Correlation analysis of gene expression levels split by conditions (fresh (**red**);

cryopreserved -80ºC: **blue**; cryopreserved liquid nitrogen: **green**). (**a-d**) Pearson's correlation analysis between 20 randomly selected fresh and cryopreserved cells displaying the correlation coefficient (r^2). (**e-h**) Distribution of Pearson's correlation coefficients (r^2) within and between processing conditions. The median coefficients are indicated. (**i-k**) Linear regression model comparing average gene expression levels of expressed genes. The coefficient of determination (r^2) is displayed. Cell lines and experiments are indicated within the figures.

Figure S11 Cell subtype analysis of HEK293 (experiment 1) cells split by fresh (**a-c**) and cryopreserved (**d-f**) cells. (**a,d**) Hierarchical clustering of single cells based on transcriptional programs (defined by gene ontology) and correlating genes [21]. Transcriptional programs and gene clusters are summarized in aspects (orange: overrepresented; green: underrepresented). Displayed are the most variable aspects (rows) and their importance (row colors). Cells are assigned to condition (fresh: red; cryopreserved: blue) and clusters. (**b,e**) A t-distributed stochastic neighbor embedding (t-SNE) representation of similarities between cells using previous defined distances and cluster identity (as in **a,d**). (**c,f**) Hierarchical cluster of single cells (as in **a,d**) displaying the 25 most variable cell cycle genes (G2/M checkpoint). Expression levels of the cell cycle signature are summarized (1st panel; orange: high, green: low) and clusters are indicated.

Figure S12 Analyses of Smart-seq2-derived single cell transcriptomes from a cryopreserved patient-derived xenograft (PDOX) tumor. (**a**) Displayed are the sequencing read distribution following RNA library preparation of full-length transcripts. Each line represents a single cell and transcript sizes are scaled from (0-100). (**b,c**) Comparative analyses of single cells from fresh (**circles**) and cryopreserved (**triangles**) samples. The displayed experiments include single cells from HEK293 cells (blue), K562 cells (orange) and the PDOX (green) sample. Gene expression variances between cells are displayed as principal component analysis (PCA, **b**) and t-distributed stochastic neighbor embedding (t-SNE, **c**) using the 100 most variable genes.

Figure S13 Cell subtype analysis of HEK293 cells analyzed by Smart-seq2 split by fresh (**a**) and

cryopreserved (**b**) cells. (**a,b**) Hierarchical clustering of single cells based on transcriptional programs (defined by gene ontology) and correlating genes [21] displaying the 25 most variable cell cycle genes (G2/M checkpoint). Expression levels of the cell cycle signature are summarized (1st panel; orange: high, green: low) and clusters are indicated. (**c**) Significantly differentially expressed genes between fresh and cryopreserved HEK293 cells ($p < 0.01$).

Figure S14 Cell subtype analysis of K562 cells analyzed by Smart-seq2 split by fresh (**a**) and cryopreserved (**b**) cells. (**a,b**) Hierarchical clustering of single cells based on transcriptional programs (defined by gene ontology) and correlating genes [21] displaying the 25 most variable cell cycle genes (G2/M checkpoint). Expression levels of the cell cycle signature are summarized (1st panel; orange: high, green: low) and clusters are indicated. (**c**) Significantly differentially expressed genes between fresh and cryopreserved K562 cells ($p < 0.01$).

Figure S15 Cell subtype analysis of fresh (**red**) and cryopreserved (**blue**) peripheral blood mononuclear cells analyzed by MARS-Seq. (**a-d**) Hierarchical clustering of single cells based on transcriptional programs (defined by gene ontology) and correlating genes [21] (as defined in Figure 5f) displaying the 25 most variable genes within the signatures for cytotoxic T-cells (a), memory T-cells (**b**), B-cells (**c**) and myeloid cells (**d**). Expression levels of all signature genes are summarized (1st panel; orange: high, green: low) and conditions (2nd panel: fresh: red; cryopreserved: blue) and clusters are indicated. The lower plots summarize the signature expression levels for each cell (dots) and cluster (box). Inferred cell types are indicated (BC: B-cells, CytoTC: cytotoxic T-cells, MemTC: memory T-cells, Myd: myeloid cells).

Figure S16 Hierarchical clustering of single cells from an ovarian tumor PDOX based on the most variable genes. Cells are assigned to conditions (2nd panel; fresh: red; cryopreserved: blue) and clusters (3rd panel). Displayed are the 25 most variable genes implicated in cluster formation. Expression levels of the genes are summarized (1st panel; orange: high, green: low).

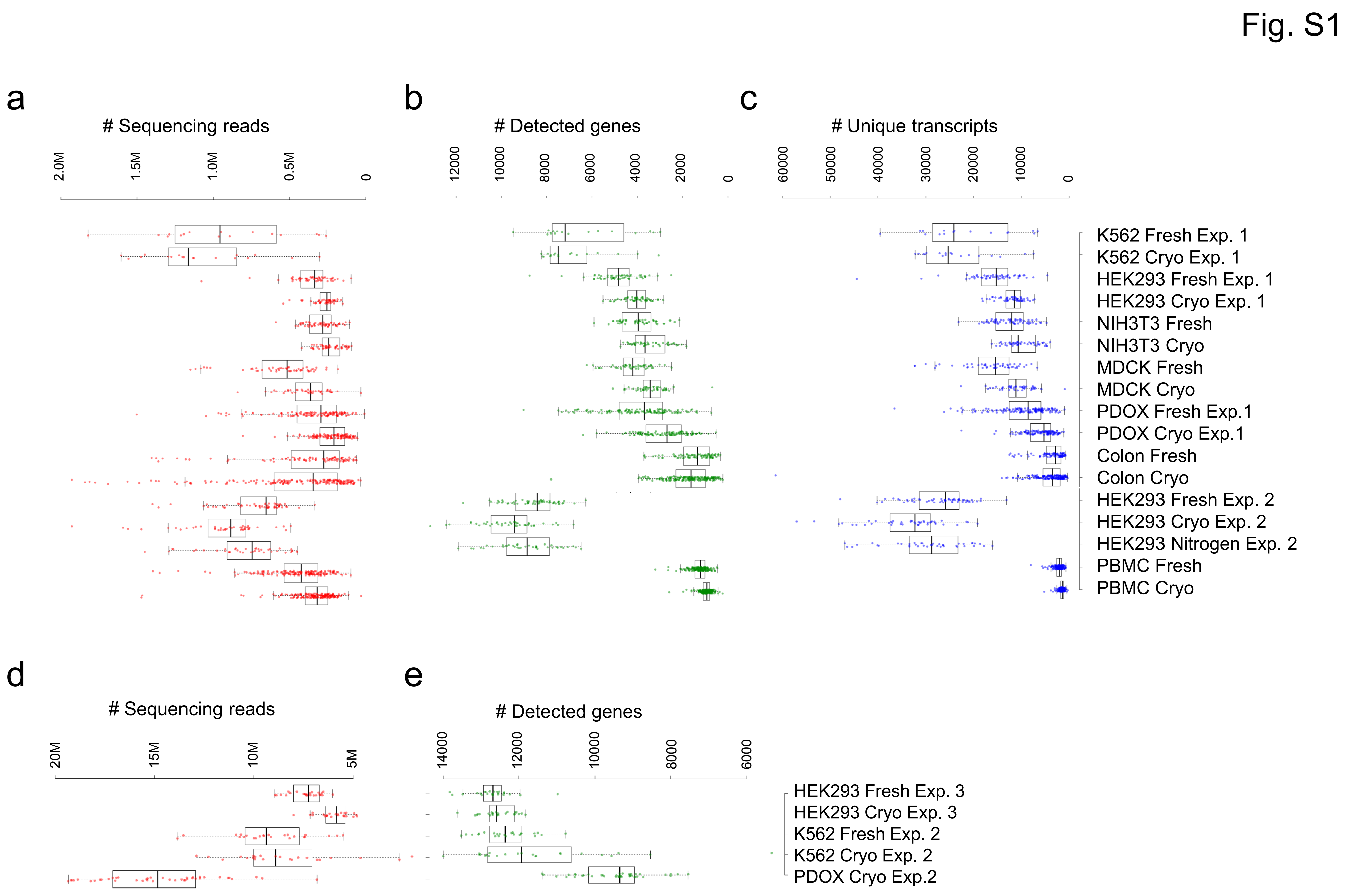
Fig. S1
a
Sequencing reads
2.0M
1.5M
1.0M
0.5M
0
b
Detected genes
12000
10000
8000
6000
4000
2000
0
c
Unique transcripts
60000
50000
40000
30000
20000
10000
0
K562 Fresh Exp. 1
K562 Cryo Exp. 1
HEK293 Fresh Exp. 1
HEK293 Cryo Exp. 1
NIH3T3 Fresh
NIH3T3 Cryo
MDCK Fresh
MDCK Cryo
PDOX Fresh Exp.1
PDOX Cryo Exp.1
Colon Fresh
Colon Cryo
HEK293 Fresh Exp. 2
HEK293 Cryo Exp. 2
HEK293 Nitrogen Exp. 2
PBMC Fresh
PBMC Cryo
d
Sequencing reads
20M
15M
10M
5M
e
Detected genes
14000
12000
10000
8000
6000
HEK293 Fresh Exp. 3
HEK293 Cryo Exp. 3
K562 Fresh Exp. 2
K562 Cryo Exp. 2
PDOX Cryo Exp.2

Fig. S2

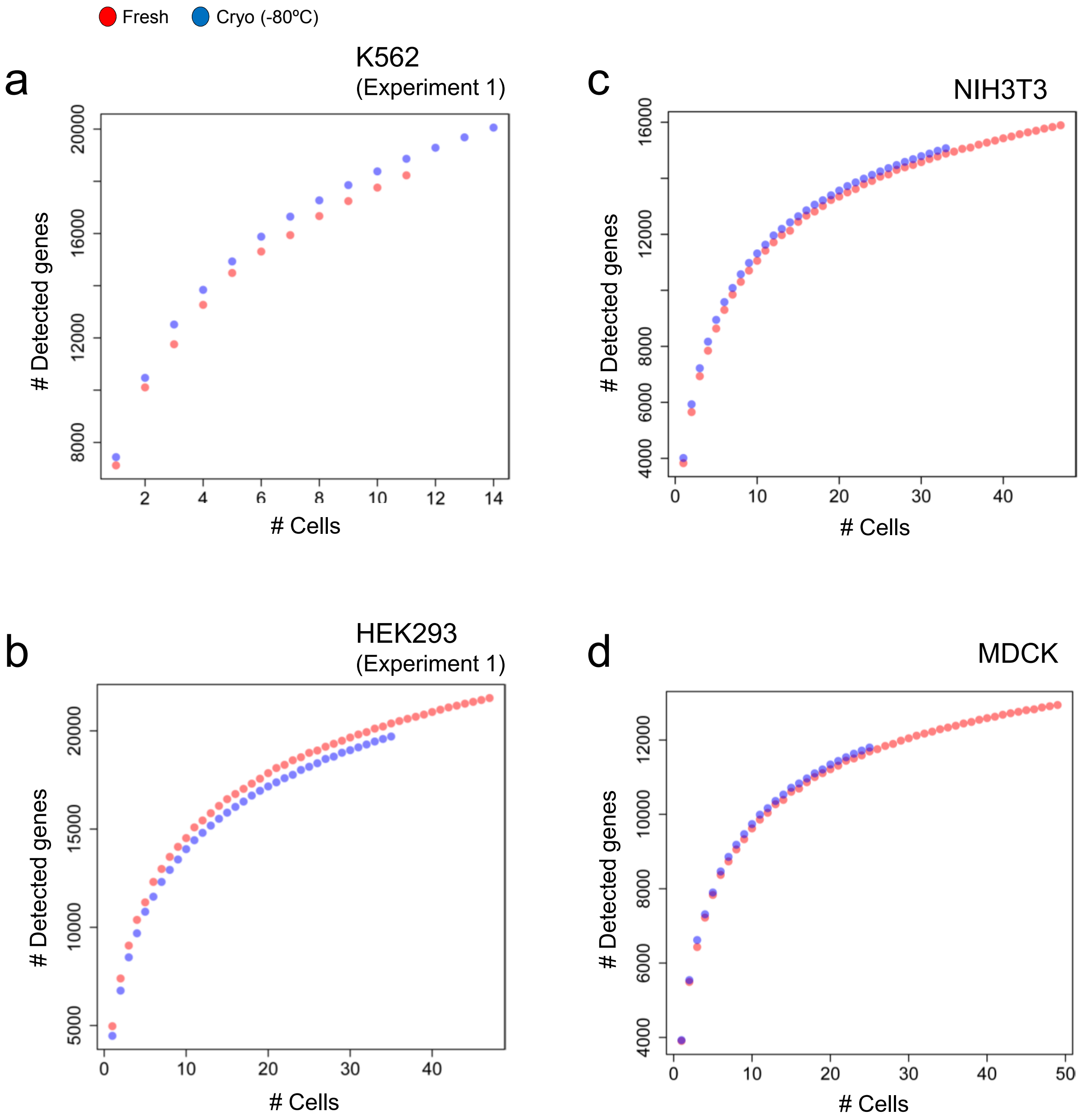
Fresh
Cryo (-80ºC)
a
K562
(Experiment 1)
Detected genes
Cells
b
HEK293
(Experiment 1)
Detected genes
Cells
c
NIH3T3
Detected genes
Cells
d
MDCK
Detected genes
Cells

Fig. S3

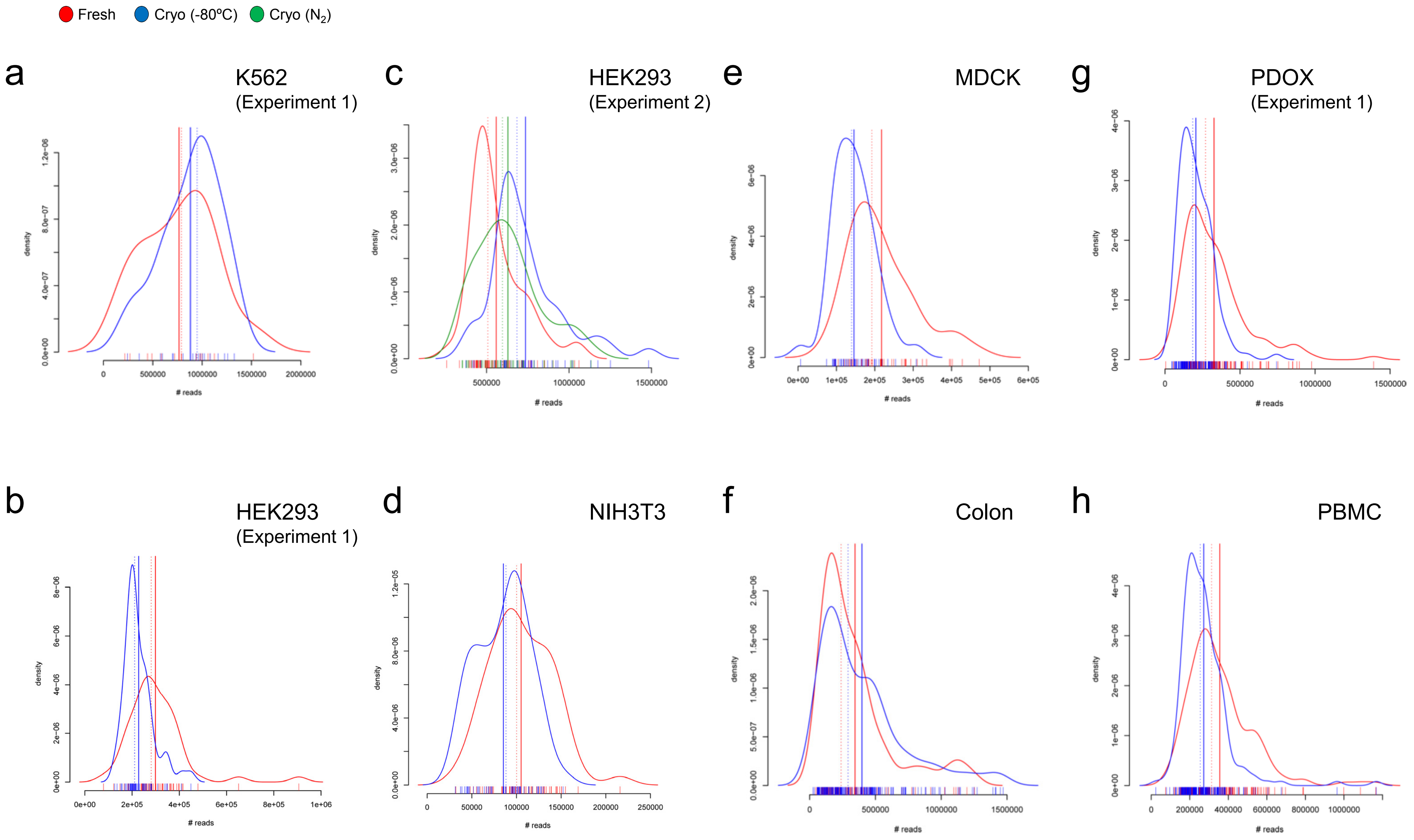

Fresh
Cryo (-80ºC)
Cryo (N2)
a
K562
(Experiment 1)
b
HEK293
(Experiment 1)
c
HEK293
(Experiment 2)
d
NIH3T3
e
MDCK
f
Colon
g
PDOX
(Experiment 1)
h
PBMC
density
reads

Fig. S4

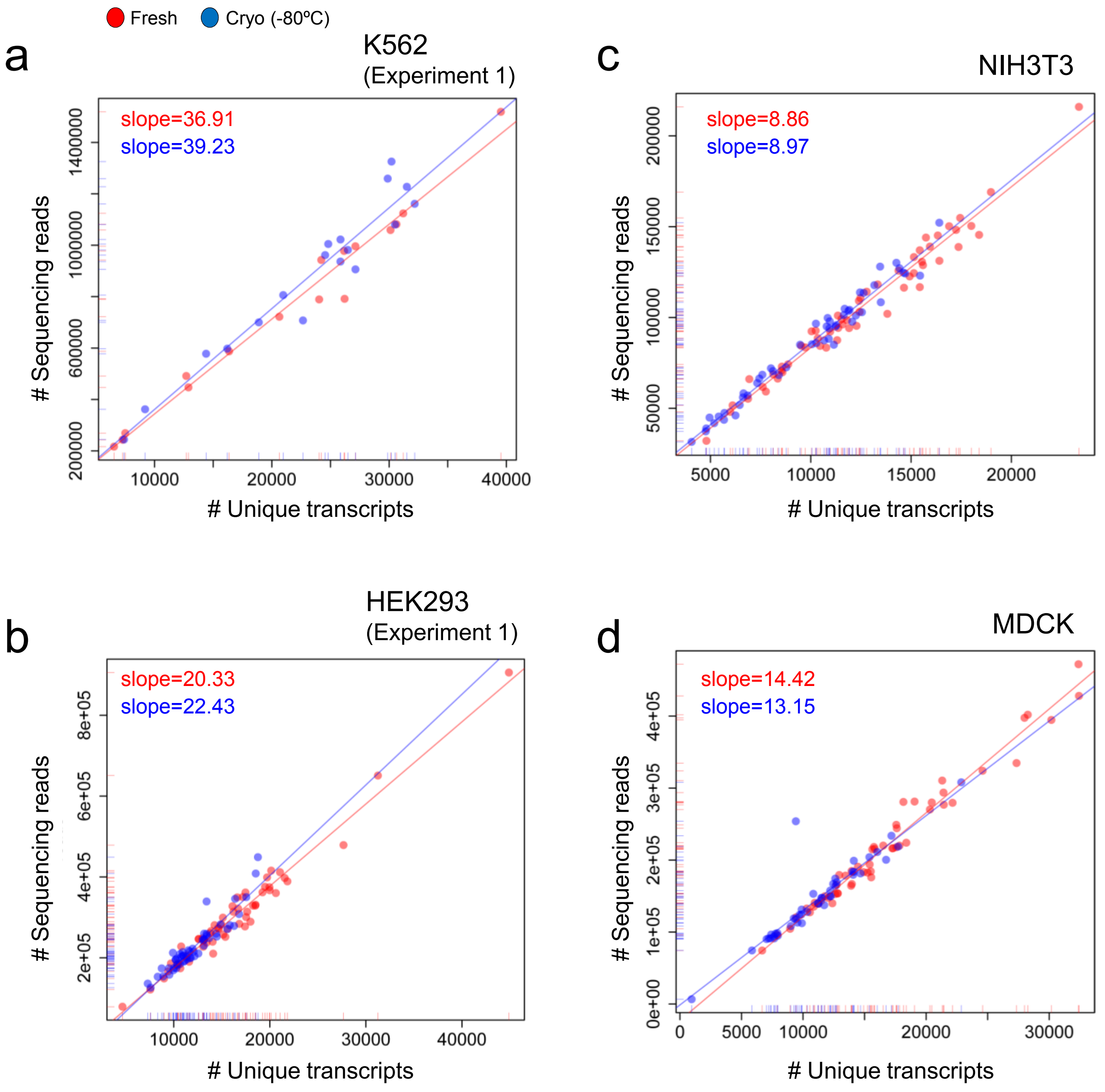
Fresh
Cryo (-80ºC)
a
K562
(Experiment 1)
slope=36.91
slope=39.23
# Sequencing reads
200000
600000
1000000
1400000
10000
20000
30000
40000
Unique transcripts
c
NIH3T3
slope=8.86
slope=8.97
# Sequencing reads
50000
100000
150000
200000
5000
10000
15000
20000
Unique transcripts
b
HEK293
(Experiment 1)
slope=20.33
slope=22.43
# Sequencing reads
2e+05
4e+05
6e+05
8e+05
10000
20000
30000
40000
Unique transcripts
d
MDCK
slope=14.42
slope=13.15
# Sequencing reads
0e+00
1e+05
2e+05
3e+05
4e+05
0
5000
10000
20000
30000
Unique transcripts

Fig. S5

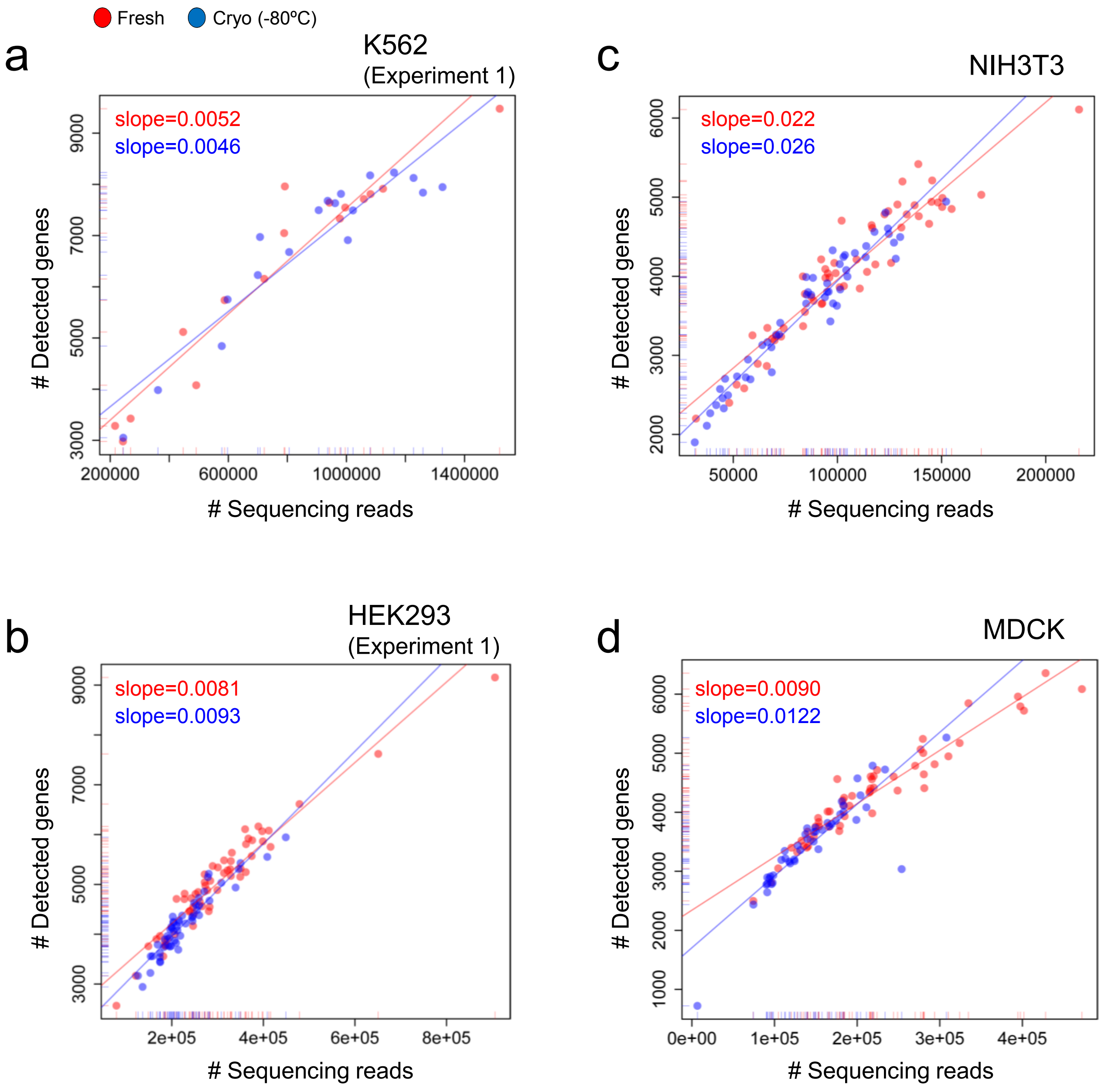
Fresh
Cryo (-80ºC)
a
K562
(Experiment 1)
slope=0.0052
slope=0.0046
# Detected genes
# Sequencing reads
3000
5000
7000
9000
200000
600000
1000000
1400000
c
NIH3T3
slope=0.022
slope=0.026
# Detected genes
# Sequencing reads
2000
3000
4000
5000
6000
50000
100000
150000
200000
b
HEK293
(Experiment 1)
slope=0.0081
slope=0.0093
# Detected genes
# Sequencing reads
3000
5000
7000
9000
2e+05
4e+05
6e+05
8e+05
d
MDCK
slope=0.0090
slope=0.0122
# Detected genes
# Sequencing reads
1000
2000
3000
4000
5000
6000
0e+00
1e+05
2e+05
3e+05
4e+05

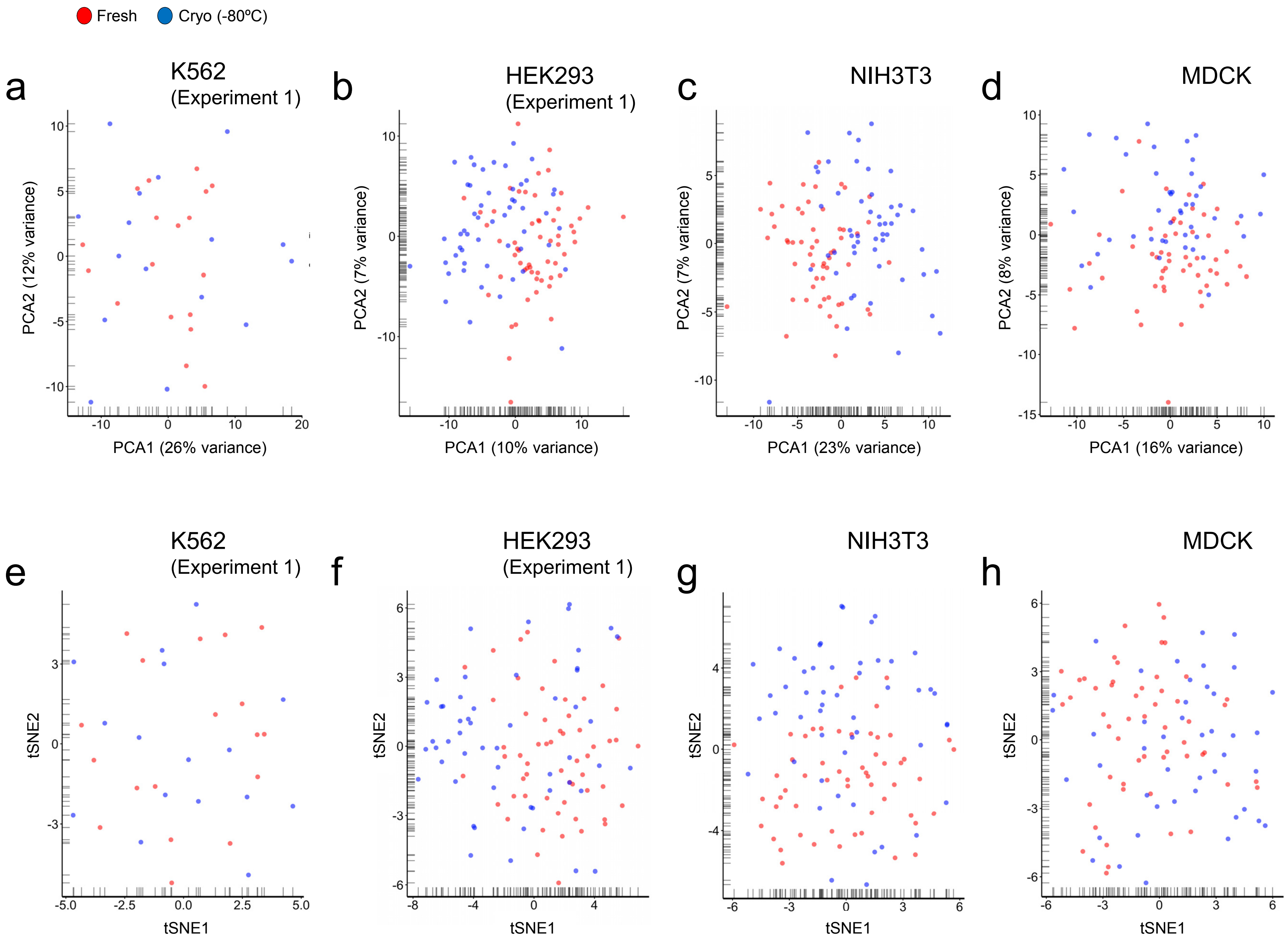
Fresh
Cryo (-80ºC)
a
K562
(Experiment 1)
PCA2 (12% variance)
PCA1 (26% variance)
b
HEK293
(Experiment 1)
PCA2 (7% variance)
PCA1 (10% variance)
c
NIH3T3
PCA2 (7% variance)
PCA1 (23% variance)
d
MDCK
PCA2 (8% variance)
PCA1 (16% variance)
e
K562
(Experiment 1)
tSNE2
tSNE1
f
HEK293
(Experiment 1)
tSNE2
tSNE1
g
NIH3T3
tSNE2
tSNE1
h
MDCK
tSNE2
tSNE1

Fig. S7

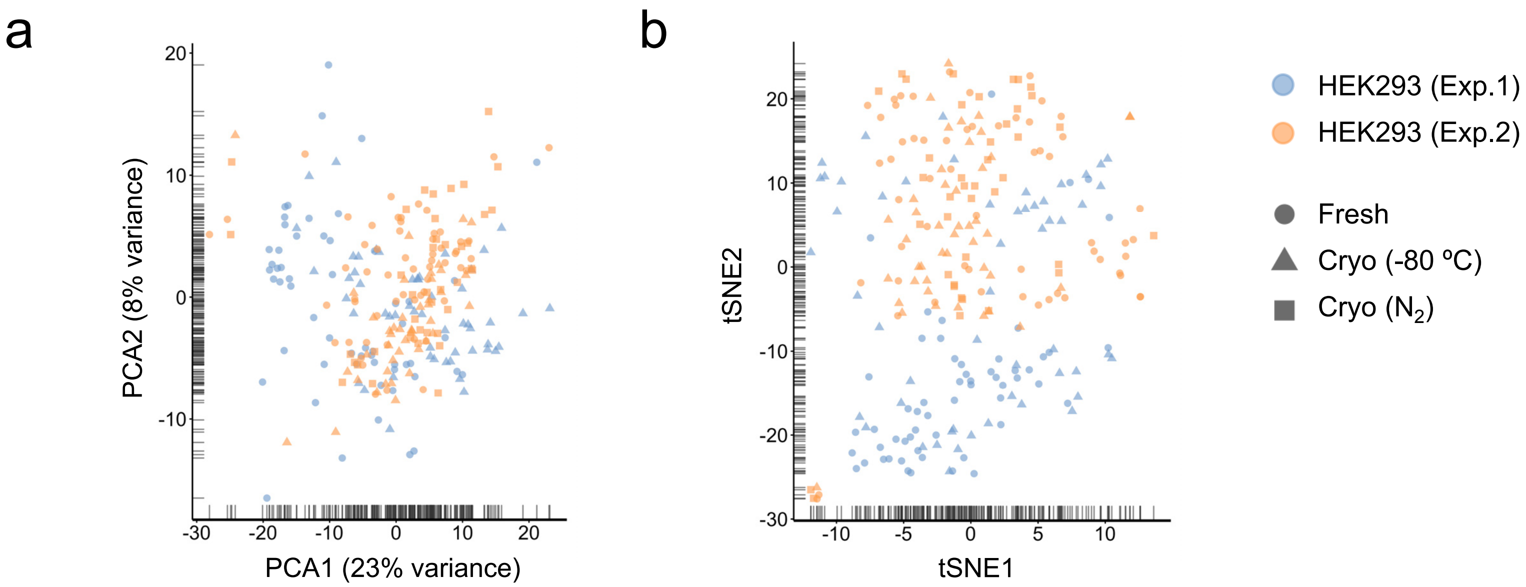
a
b
PCA2 (8% variance)
PCA1 (23% variance)
20
10
0
-10
-30
-20
-10
0
10
20
tSNE2
tSNE1
20
10
0
-10
-20
-30
-10
-5
0
5
10
HEK293 (Exp.1)
HEK293 (Exp.2)
Fresh
Cryo (-80 ºC)
Cryo (N_2)

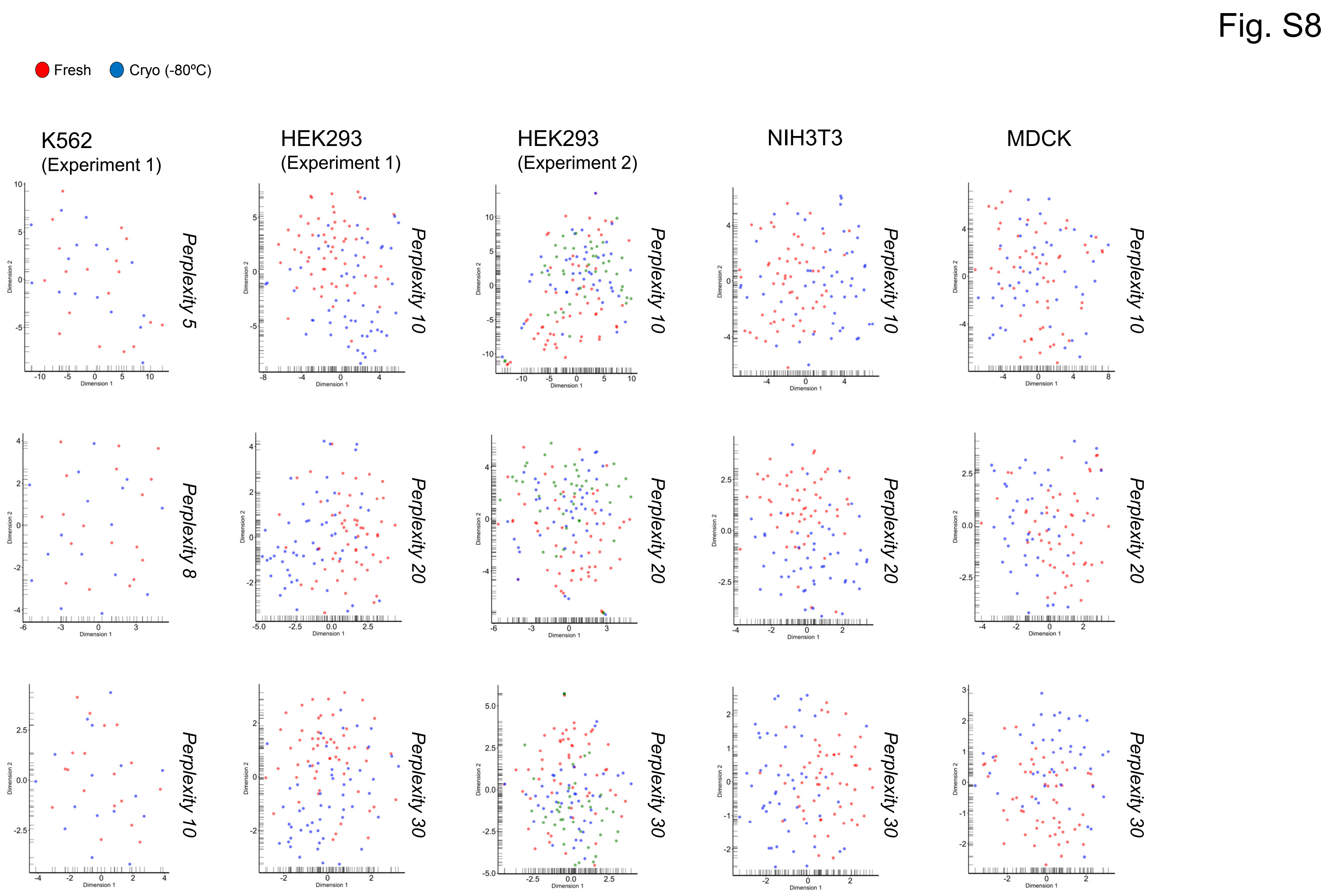
Fig. S8
Fresh
Cryo (-80ºC)
K562
(Experiment 1)
HEK293
(Experiment 1)
HEK293
(Experiment 2)
NIH3T3
MDCK
Perplexity 5
Perplexity 10
Perplexity 8
Perplexity 20
Perplexity 30
Dimension 1
Dimension 2

Fig. S9

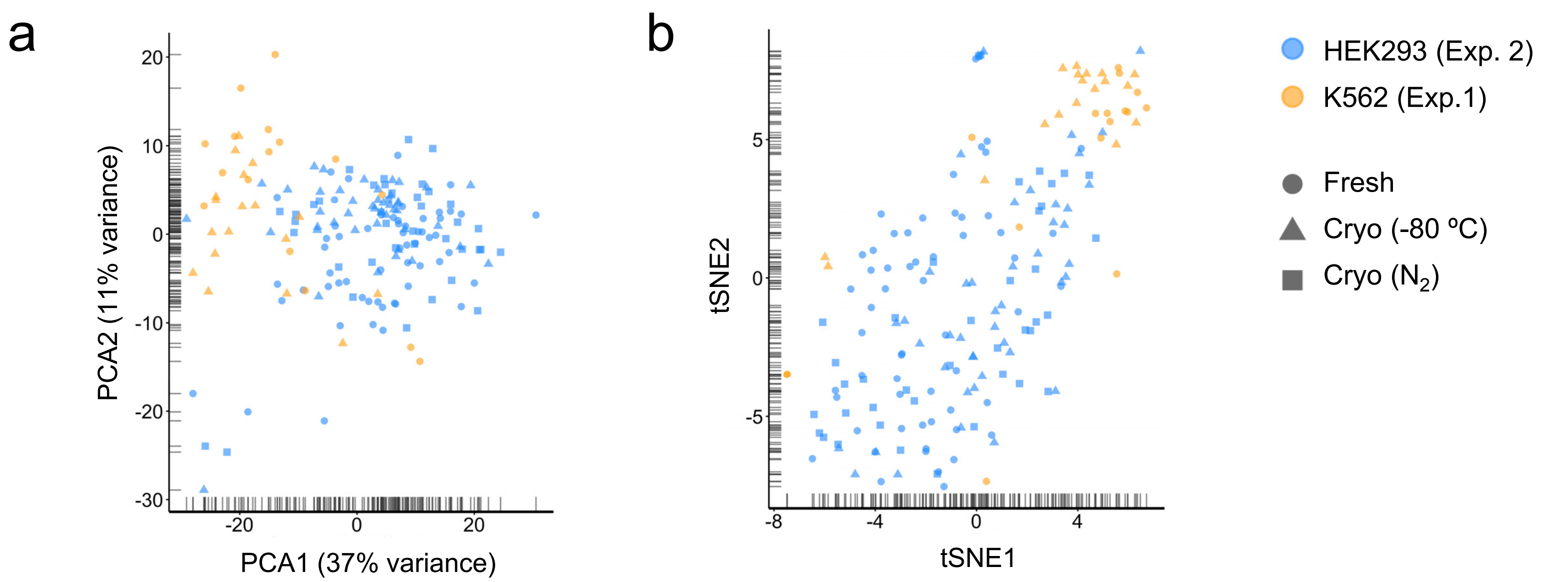
a
PCA2 (11% variance)
PCA1 (37% variance)
20
10
0
-10
-20
-30
-20
0
20
b
tSNE2
tSNE1
5
0
-5
-8
-4
0
4
HEK293 (Exp. 2)
K562 (Exp.1)
Fresh
Cryo (-80 ºC)
Cryo (N2)

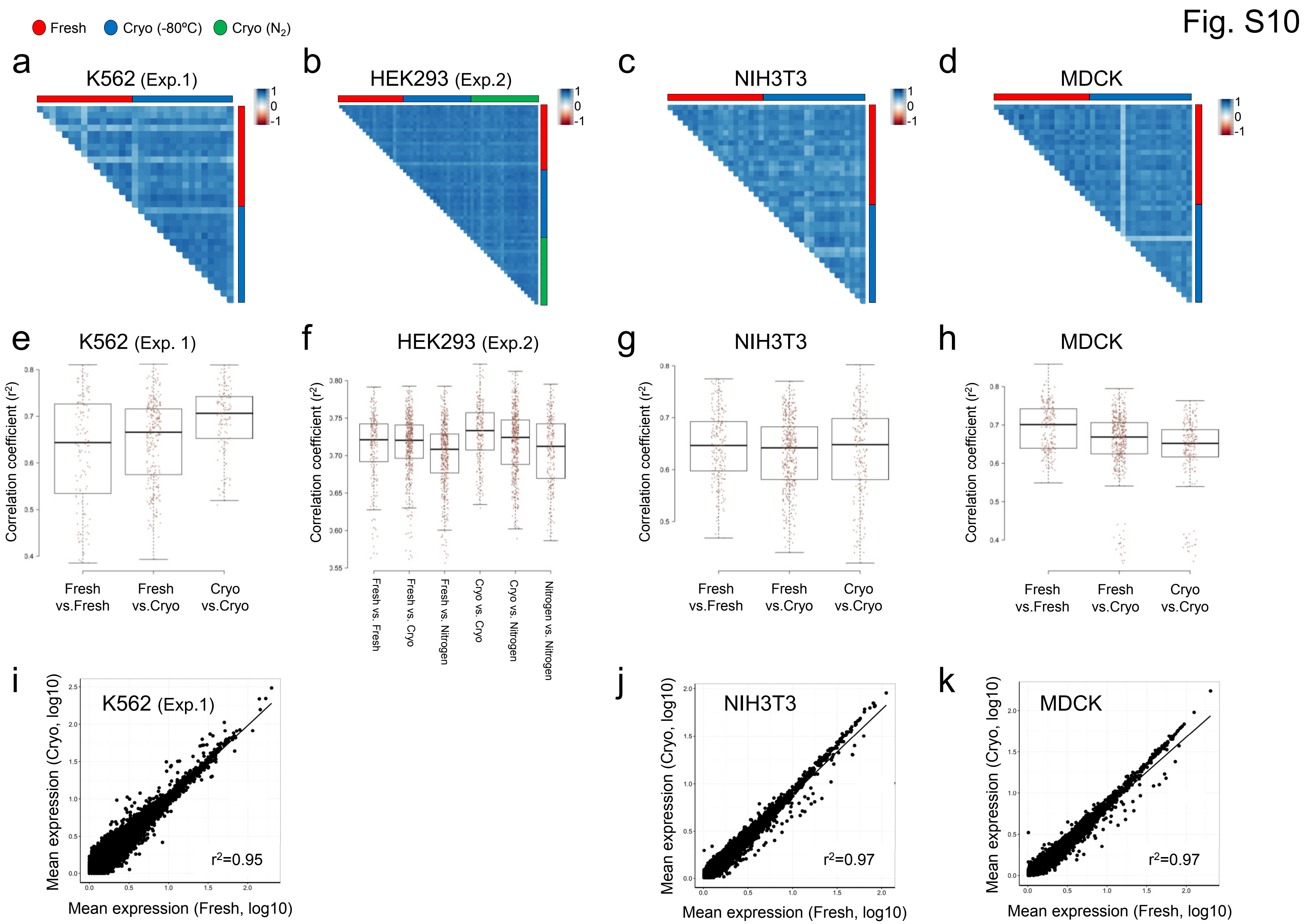

Fig. S10
Fresh
Cryo (-80ºC)
Cryo (N2)
a
K562 (Exp.1)
b
HEK293 (Exp.2)
c
NIH3T3
d
MDCK
e
K562 (Exp. 1)
f
HEK293 (Exp.2)
g
NIH3T3
h
MDCK
Correlation coefficient (r2)
Fresh vs.Fresh
Fresh vs.Cryo
Cryo vs.Cryo
Fresh vs. Fresh
Fresh vs. Cryo
Fresh vs. Nitrogen
Cryo vs. Cryo
Cryo vs. Nitrogen
Nitrogen vs. Nitrogen
i
K562 (Exp.1)
r2=0.95
j
NIH3T3
r2=0.97
k
MDCK
r2=0.97
Mean expression (Cryo, log10)
Mean expression (Fresh, log10)

Fig. S11

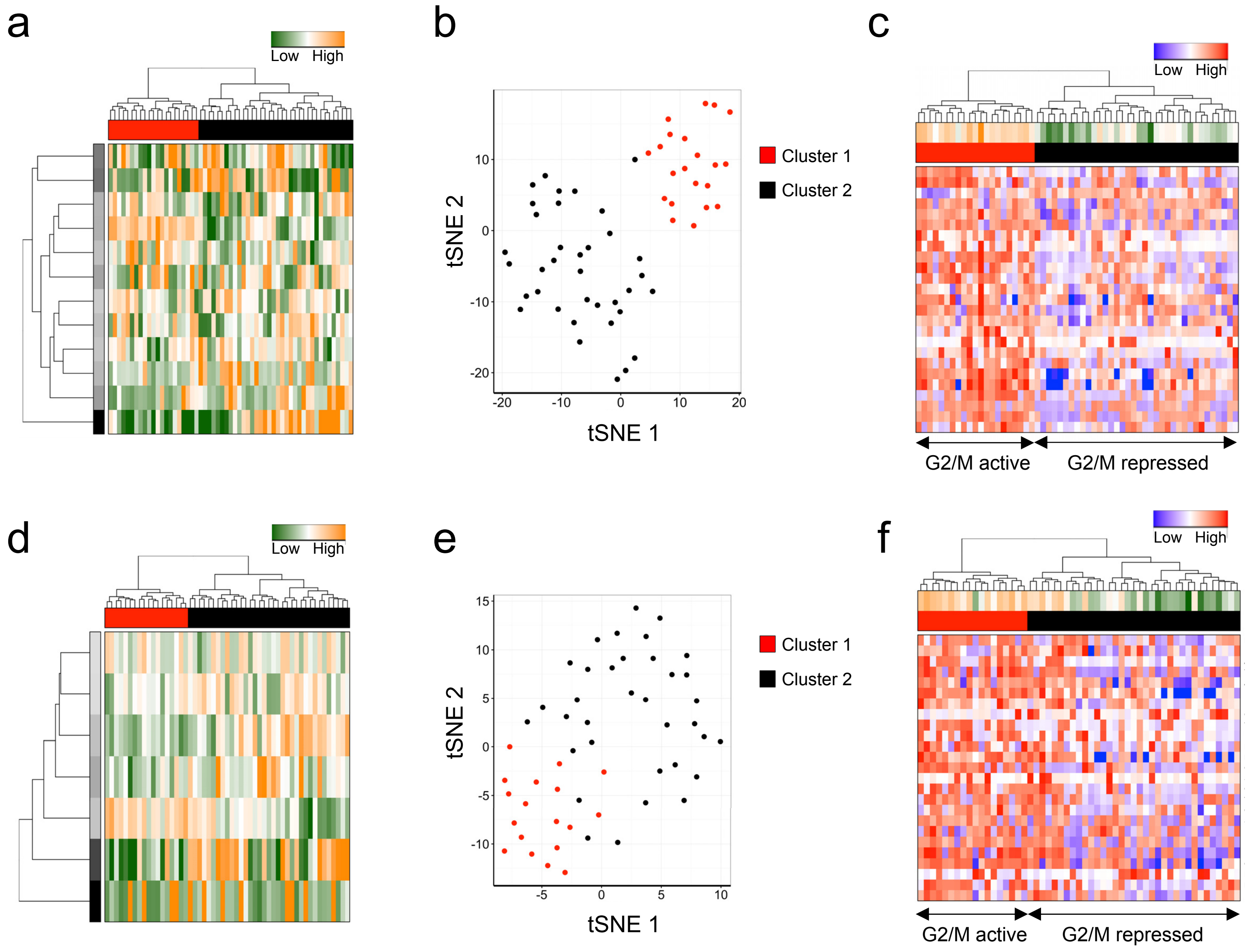
a
Low High
b
tSNE 2
tSNE 1
Cluster 1
Cluster 2
c
Low High
G2/M active
G2/M repressed
d
Low High
e
tSNE 2
tSNE 1
Cluster 1
Cluster 2
f
Low High
G2/M active
G2/M repressed

Fig. S12

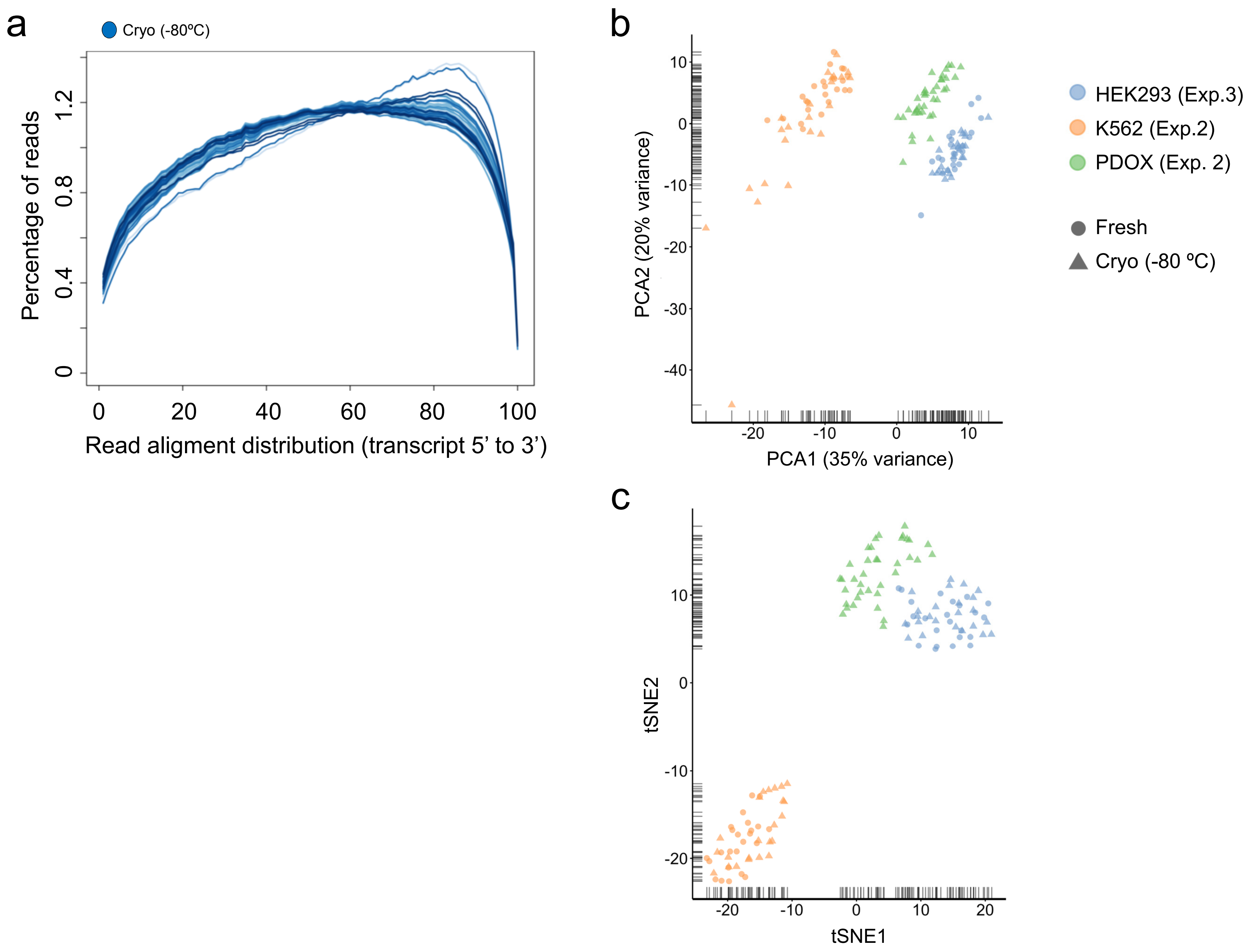

a
Cryo (-80ºC)
Percentage of reads
0
0.4
0.8
1.2
0
20
40
60
80
100
Read aligment distribution (transcript 5' to 3')
b
PCA2 (20% variance)
10
0
-10
-20
-30
-40
-20
-10
0
10
PCA1 (35% variance)
HEK293 (Exp.3)
K562 (Exp.2)
PDOX (Exp. 2)
Fresh
Cryo (-80 ºC)
c
tSNE2
10
0
-10
-20
-20
-10
0
10
20
tSNE1

Fig. S13

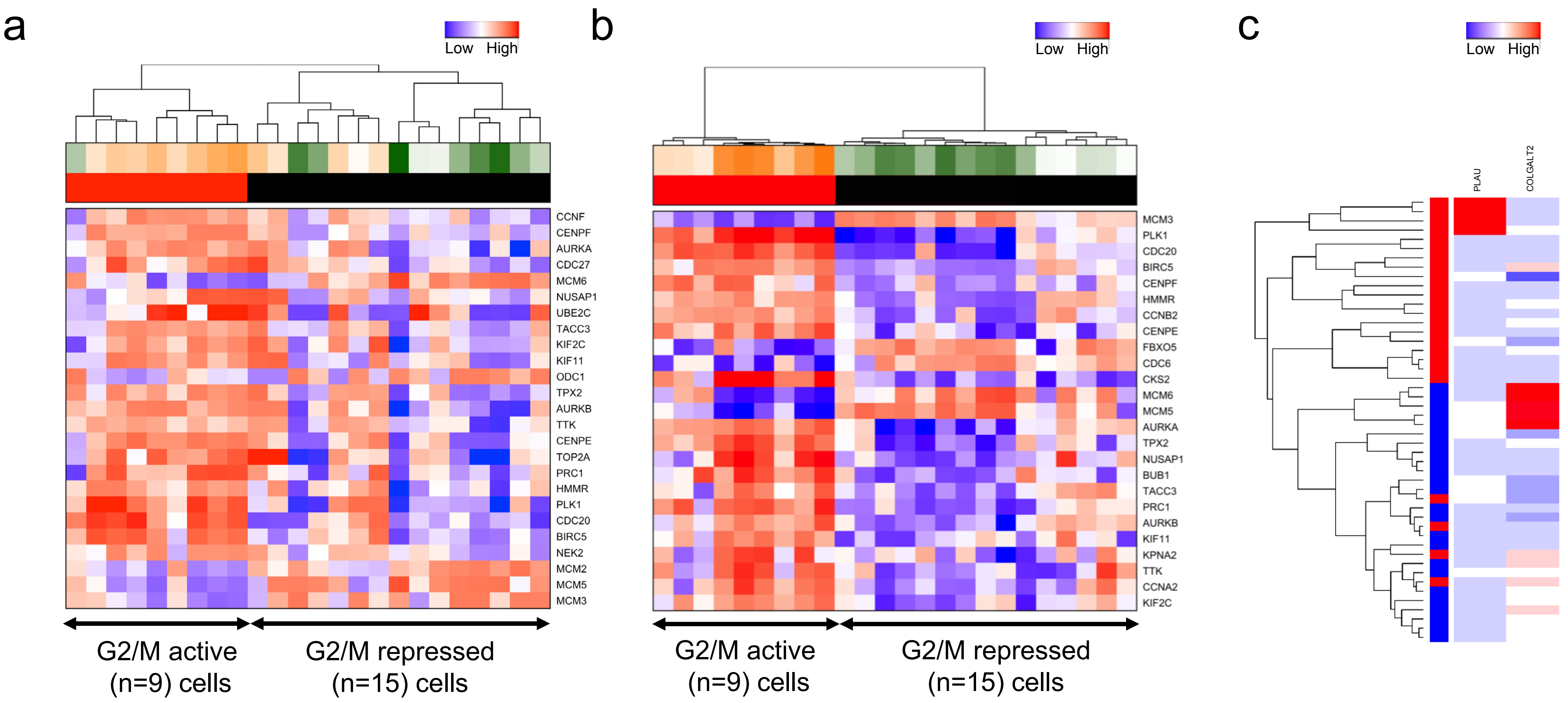

a
Low High
CCNF
CENPF
AURKA
CDC27
MCM6
NUSAP1
UBE2C
TACC3
KIF2C
KIF11
ODC1
TPX2
AURKB
TTK
CENPE
TOP2A
PRC1
HMMR
PLK1
CDC20
BIRC5
NEK2
MCM2
MCM5
MCM3
G2/M active (n=9) cells
G2/M repressed (n=15) cells
b
Low High
MCM3
PLK1
CDC20
BIRC5
CENPF
HMMR
CCNB2
CENPE
FBXO5
CDC6
CKS2
MCM6
MCM5
AURKA
TPX2
NUSAP1
BUB1
TACC3
PRC1
AURKB
KIF11
KPNA2
TTK
CCNA2
KIF2C
G2/M active (n=9) cells
G2/M repressed (n=15) cells
c
Low High
PLAU
COLGALT2

Fig. S14

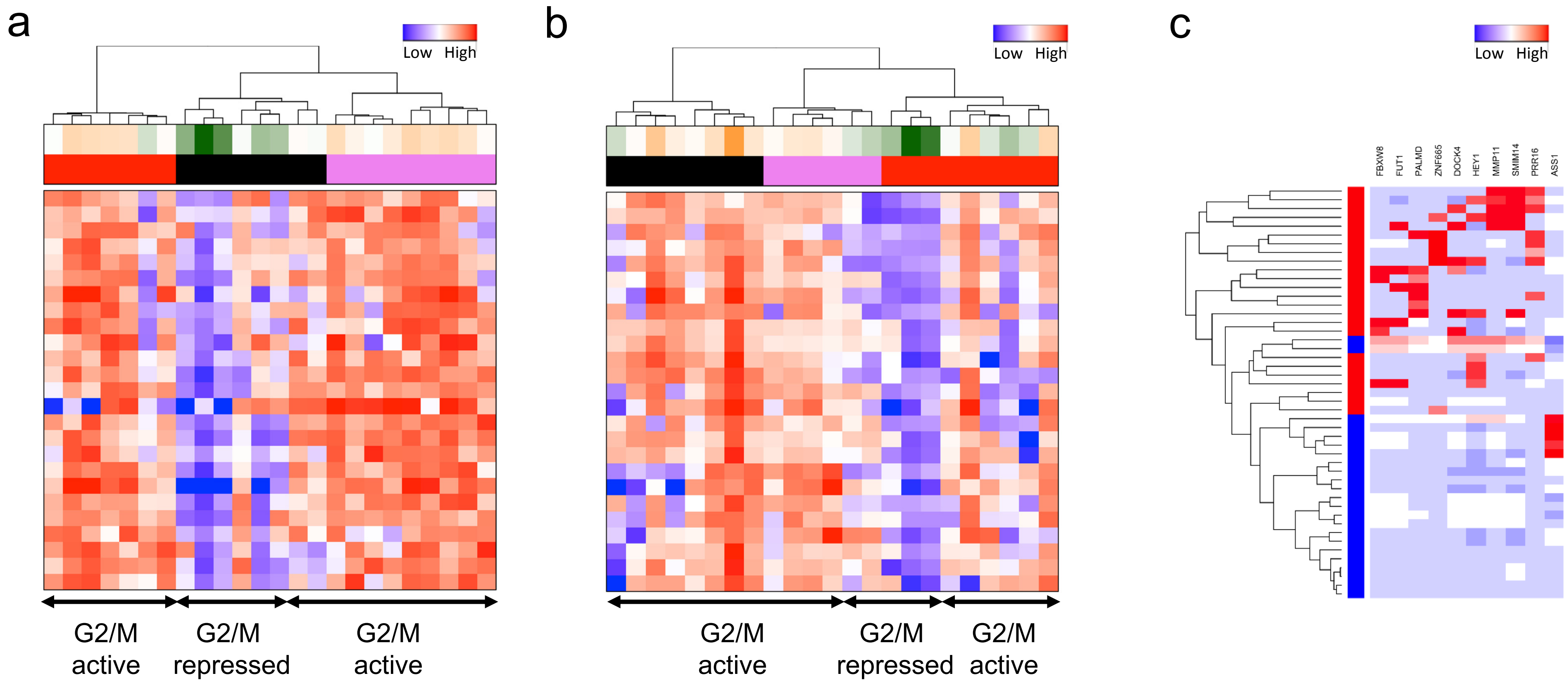
a
Low High
G2/M
active
G2/M
repressed
G2/M
active
b
Low High
G2/M
active
G2/M
repressed
G2/M
active
c
Low High
FBXW8
FUT1
PALMD
ZNF665
DOCK4
HEY1
MMP11
SMIM14
PRR16
ASS1

Fig. S15

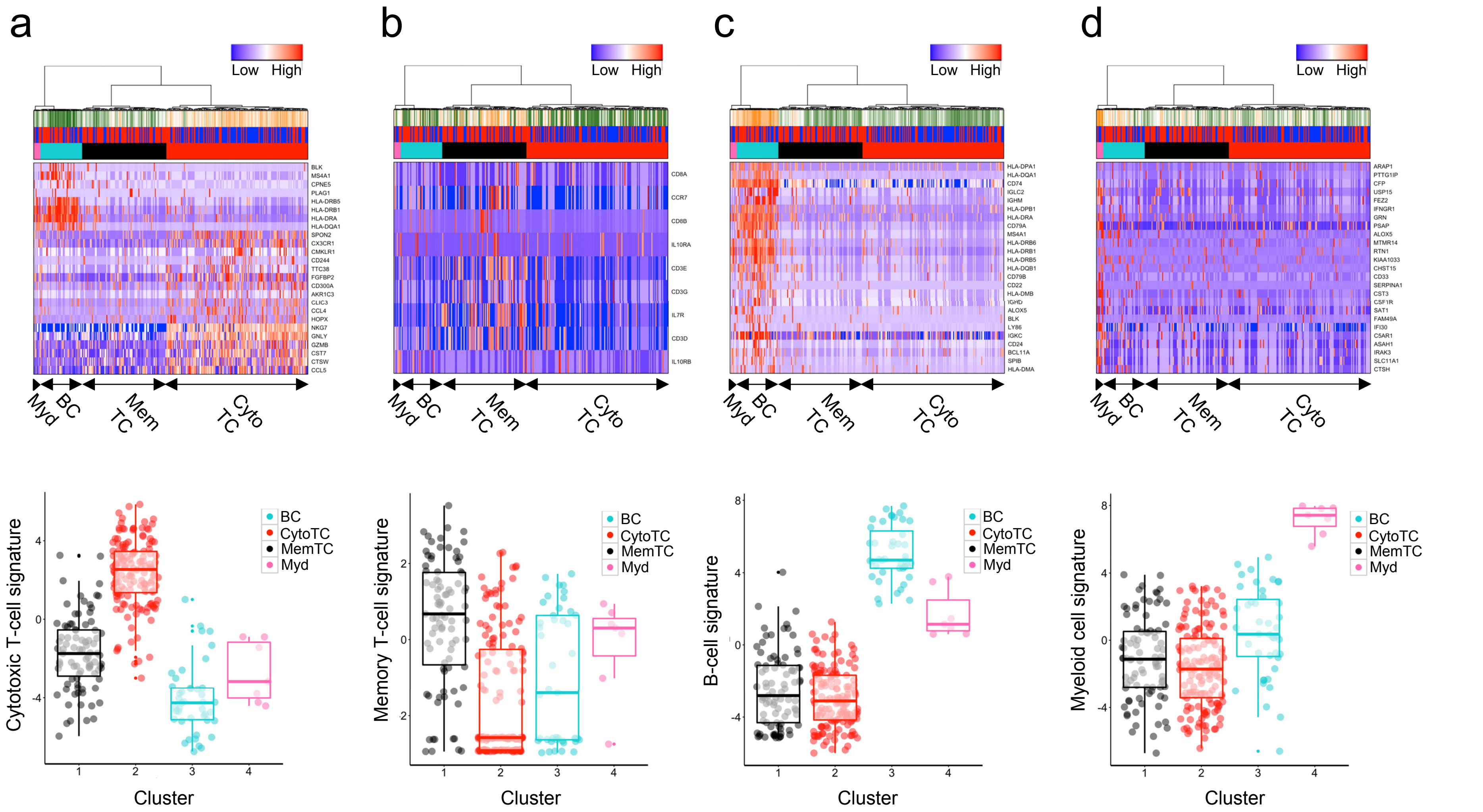

a
b
c
d
Low
High
Myd
BC
Mem TC
Cyto TC
Cytotoxic T-cell signature
Memory T-cell signature
B-cell signature
Myeloid cell signature
Cluster
BC
CytoTC
MemTC
Myd

Fig. S16

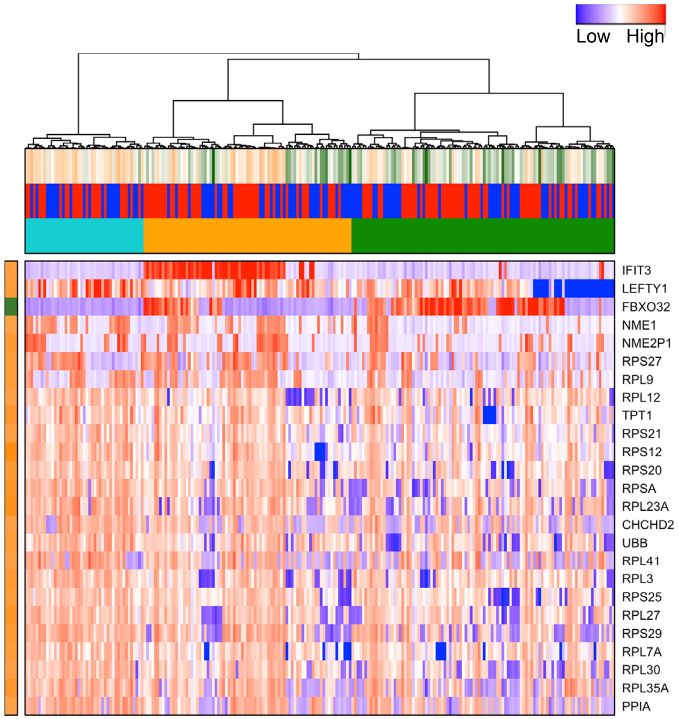

Low
High
IFIT3
LEFTY1
FBXO32
NME1
NME2P1
RPS27
RPL9
RPL12
TPT1
RPS21
RPS12
RPS20
RPSA
RPL23A
CHCHD2
UBB
RPL41
RPL3
RPS25
RPL27
RPS29
RPL7A
RPL30
RPL35A
PPIA
