# Supplementary material for: Single-cell transcriptome conservation in cryopreserved cells and tissues
Source: Genome Biol. 2017 Mar 1;18:45. doi: 10.1186/s13059-017-1171-9 (PMC5333448; doi:10.1186/s13059-017-1171-9)
Supplement: Additional file 2: Tables S1–9. — Table S1. Overview table of experiments. Table S2. Differential gene expression between fresh and cryopreserved K562 cells. (MARS-Seq; top 40 genes). Table S3. Differential gene expression between fresh and cryopreserved HEK293 cells. (MARS-Seq; top 40 genes). Table S4. Differential gene expression between fresh and cryopreserved NIH3T3 cells. (MARS-Seq; top 40 genes). Table S5. Differential gene expression between fresh and cryopreserved MDCK cells. (MARS-Seq; top 40 genes). Table 6: Differential gene expression between fresh and cryopreserved HEK293 cells. (SMARTseq2; top 40 genes). Table 7: Differential gene expression between fresh and cryopreserved K562 cells. (SMARTseq2; top 40 genes). Table S8: Differential gene expression between fresh and cryopreserved PBMC. (MARS-Seq; top 40 genes). Table S9: Differential gene expression between a fresh and cryopreserved PDOX. (MARSseq; top 40 genes). (PDF 40 kb) [file 13059_2017_1171_MOESM2_ESM.pdf]

Table S1: Overview table of experiments.

Cells/tissue	Type	Species	Method	No.	Condition	Storage	Cells	Empty wells (%)	Cells <100K reads (%)	Cells filtered (%)
K562	Cell line	Human	MARS-Seq	1	Fresh	N/A	**16**	27	0	0
K562	Cell line	Human	MARS-Seq	1	Cryopreserved	-80°C	**18**	18	0	0
K562	Cell line	Human	SMARTseq2	2	Fresh	N/A	**24**	N/A	0	0
K562	Cell line	Human	SMARTseq2	2	Cryopreserved	-80°C	**23**	N/A	0	0
HEK293	Cell line	Human	MARS-Seq	1	Fresh	N/A	**57**	5	2	0
HEK293	Cell line	Human	MARS-Seq	1	Cryopreserved	-80°C	**53**	4	0	0
HEK293	Cell line	Human	MARS-Seq	2	Fresh	N/A	**58**	8	0	0
HEK293	Cell line	Human	MARS-Seq	2	Cryopreserved	-80°C	**44**	30	0	0
HEK293	Cell line	Human	MARS-Seq	2	Cryopreserved	Liquid N2	**40**	38	0	0
HEK293	Cell line	Human	SMARTseq2	3	Fresh	N/A	**24**	N/A	0	0
HEK293	Cell line	Human	SMARTseq2	3	Cryopreserved	-80°C	**24**	N/A	0	0
NIH3T3	Cell line	Mouse	MARS-Seq	1	Fresh	N/A	**56**	7	0	0
NIH3T3	Cell line	Mouse	MARS-Seq	1	Cryopreserved	-80°C	**51**	7	2	0
MDCK	Cell line	Dog	MARS-Seq	1	Fresh	N/A	**54**	10	0	0
MDCK	Cell line	Dog	MARS-Seq	1	Cryopreserved	-80°C	**45**	18	2	2
Mouse colon	Primary	Mouse	MARS-Seq	1	Fresh	N/A	**88**	7	5	18
Mouse colon	Primary	Mouse	MARS-Seq	1	Cryopreserved	-80°C	**181**	4	7	15
Ovarian tumor	Xenograft	Human	MARS-Seq	1	Fresh	N/A	**132**	21	4	5
Ovarian tumor	Xenograft	Human	MARS-Seq	1	Cryopreserved	-80°C	**115**	25	9	15
Lung tumor	Xenograft	Human	SMARTseq2	1	Cryopreserved	-80°C	**42**	N/A	0	0
PBMC	Primary	Human	MARS-Seq	1	Fresh	N/A	**161**	16	1	8
PBMC	Primary	Human	MARS-Seq	1	Cryopreserved	-80°C	**180**	6	1	22
**TOTAL**							**1486**		**3**	**8**
**TOTAL (fresh)**							**670**		**2**	**5**
**TOTAL (cryopreserved)**							**816**		**3**	**11**

Table S2: Differential gene expression between fresh and cryopreserved K562 cells. (MARS-Seq; top 40 genes).

Gene	Lower bound	log2 fold change	Upper bound	p-value	Adj p-value
DHFR	-2,84	-1,85	-0,92	6,72E-05	**6,49E-01**
MT-ATP6	-2,48	-1,49	-0,69	1,45E-04	**7,00E-01**
MTRNR2L8	-2,74	-1,82	-0,83	3,31E-04	**1,00E+00**
MT-ND2	-2,28	-1,49	-0,63	5,47E-04	**1,00E+00**
MTRNR2L12	-3,40	-2,38	-1,02	5,96E-04	**1,00E+00**
ANKRD30BL	-1,78	-1,09	-0,46	1,33E-03	**1,00E+00**
MT-ND5	-1,78	-1,09	-0,43	1,57E-03	**1,00E+00**
MT-ND3	-3,73	-2,28	-0,86	1,57E-03	**1,00E+00**
MTATP6P1	-3,20	-2,01	-0,79	1,94E-03	**1,00E+00**
RP11-352E6.2	-3,20	-1,65	-0,63	2,27E-03	**1,00E+00**
MT-CO2	-2,18	-1,32	-0,50	2,36E-03	**1,00E+00**
MT-CYB	-1,85	-1,12	-0,40	2,45E-03	**1,00E+00**
MTCO2P2	-2,84	-1,68	-0,63	2,71E-03	**1,00E+00**
MT-TI	-3,53	-2,08	-0,69	2,78E-03	**1,00E+00**
MTND5P11	-2,64	-1,55	-0,50	4,28E-03	**1,00E+00**
SMAD2	-2,77	-1,62	-0,53	4,46E-03	**1,00E+00**
RP11-475J5.4	-3,10	-1,82	-0,53	6,28E-03	**1,00E+00**
MT-ND4L	-3,10	-1,88	-0,56	6,31E-03	**1,00E+00**
MTND1P23	-1,58	-0,86	-0,23	7,92E-03	**1,00E+00**
PLCB4	-3,23	-2,08	-0,59	7,96E-03	**1,00E+00**
HIST1H1E	-2,38	-1,39	-0,40	8,60E-03	**1,00E+00**
MTND1P27	-1,98	-1,06	-0,26	8,78E-03	**1,00E+00**
ID3	0,46	1,95	2,94	8,96E-03	**1,00E+00**
MT-ND4	-1,42	-0,89	-0,23	9,41E-03	**1,00E+00**
PPM1L	-3,23	-1,95	-0,46	1,27E-02	**1,00E+00**
MTRNR2L1	-1,72	-0,96	-0,20	1,58E-02	**1,00E+00**
ECHDC3	-2,87	-1,72	-0,36	1,59E-02	**1,00E+00**
ELOVL7	-2,84	-1,49	-0,30	1,62E-02	**1,00E+00**
MTRNR2L6	-2,84	-1,58	-0,30	1,75E-02	**1,00E+00**
DDIT4	0,36	2,05	3,33	1,82E-02	**1,00E+00**
MT-ATP8	-2,11	-1,16	-0,20	1,88E-02	**1,00E+00**
RP11-120M18.2	-2,81	-1,68	-0,33	1,96E-02	**1,00E+00**
MTND2P28	-2,57	-1,39	-0,20	2,34E-02	**1,00E+00**
AC105402.4	-2,71	-1,52	-0,20	2,39E-02	**1,00E+00**
RP11-475J5.8	-1,72	-0,89	-0,13	2,56E-02	**1,00E+00**
EXOSC3	-2,18	-1,16	-0,17	2,71E-02	**1,00E+00**
DYNC2H1	-2,81	-1,68	-0,17	3,12E-02	**1,00E+00**
TSC22D4	0,10	1,16	2,81	3,21E-02	**1,00E+00**
MTND4P12	-1,95	-0,99	-0,10	3,29E-02	**1,00E+00**
IER2	-1,98	-1,02	-0,10	3,50E-02	**1,00E+00**

Table S3: Differential gene expression between fresh and cryopreserved HEK293 cells. (MARS-Seq; top 40 genes).

Gene	Lower bound	log2 fold change	Upper bound	p-value	Adj p-value
RP11-849F2.7	0,89	1,72	2,58	2,67E-05	**1,19E-01**
RPL26	0,89	1,72	2,58	2,76E-05	**1,19E-01**
RPL9	0,79	1,56	2,35	3,14E-05	**1,19E-01**
RPS24	0,96	1,92	2,78	3,84E-05	**1,19E-01**
RP3-417G15.1	1,03	2,09	3,05	6,22E-05	**1,54E-01**
RPL26P19	0,83	1,75	2,65	1,00E-04	**2,07E-01**
MT-RNR1	1,22	2,55	3,61	1,23E-04	**2,18E-01**
MT-ND2	0,73	1,72	2,71	3,09E-04	**4,77E-01**
MT-CYB	0,63	1,56	2,55	6,28E-04	**8,43E-01**
RP13-258O15.1	0,66	1,62	2,58	6,82E-04	**8,43E-01**
MTRNR2L12	0,89	2,25	3,61	7,80E-04	**8,44E-01**
MT-ATP6	0,60	1,59	2,58	8,20E-04	**8,44E-01**
MTATP6P1	0,66	1,82	2,98	1,26E-03	**1,00E+00**
RPS27	0,53	1,56	2,45	2,13E-03	**1,00E+00**
MT-CO2	0,50	1,49	2,55	2,31E-03	**1,00E+00**
DHFR	0,50	1,59	2,58	3,75E-03	**1,00E+00**
XIST	0,33	1,13	1,85	4,11E-03	**1,00E+00**
RPS24P17	0,53	1,75	2,85	4,65E-03	**1,00E+00**
RPL9P9	0,30	1,03	1,79	4,70E-03	**1,00E+00**
RPS24P8	0,46	1,62	2,71	5,51E-03	**1,00E+00**
NPM1	0,20	0,79	1,36	5,84E-03	**1,00E+00**
MTCO2P2	0,46	1,89	3,21	7,49E-03	**1,00E+00**
SMAD2	0,40	1,72	2,98	8,36E-03	**1,00E+00**
PTMAP2	0,23	0,96	1,62	9,24E-03	**1,00E+00**
EPB42	0,33	1,39	2,45	9,52E-03	**1,00E+00**
RPS29	0,17	0,83	1,46	1,12E-02	**1,00E+00**
SLC12A3	0,33	1,59	2,78	1,13E-02	**1,00E+00**
MTRNR2L8	0,30	1,62	2,95	1,52E-02	**1,00E+00**
NPM1P27	0,13	0,83	1,49	1,66E-02	**1,00E+00**
RP11-507E23.1	0,23	1,39	2,45	1,67E-02	**1,00E+00**
LDHB	0,10	0,73	1,36	1,79E-02	**1,00E+00**
NUCKS1	0,13	0,96	1,72	1,84E-02	**1,00E+00**
PRDX1	0,07	0,73	1,36	2,30E-02	**1,00E+00**
FAM172A	0,07	0,70	1,29	2,47E-02	**1,00E+00**
RPL34	0,10	0,96	1,79	2,50E-02	**1,00E+00**
PTMAP5	0,07	0,70	1,29	2,50E-02	**1,00E+00**
MTRNR2L1	0,13	1,26	2,45	2,63E-02	**1,00E+00**
SLC26A3	0,17	1,52	2,88	2,75E-02	**1,00E+00**
MT-ND4	0,07	0,76	1,49	2,85E-02	**1,00E+00**
BAIAP2L1	0,07	0,89	1,62	2,93E-02	**1,00E+00**

Table S4: Differential gene expression between fresh and cryopreserved NIH3T3 cells. (MARS-Seq; top 40 genes).

Gene	Lower bound	log2 fold change	Upper bound	p-value	Adj p-value
Rps4x	0,85	1,81	2,70	1,09E-04	1,00E+00
Gm10925	0,68	2,01	3,38	2,30E-03	1,00E+00
Gm9294	0,51	1,54	2,49	2,98E-03	1,00E+00
Gm28661	0,55	2,05	3,55	6,55E-03	1,00E+00
Rps25	0,34	1,30	2,19	6,87E-03	1,00E+00
B2m	0,31	1,23	2,19	7,74E-03	1,00E+00
mt-Atp6	0,48	1,91	3,41	7,76E-03	1,00E+00
Npm1	0,27	1,23	2,15	8,39E-03	1,00E+00
mt-Nd2	0,34	1,54	2,77	8,93E-03	1,00E+00
mt-Co2	0,44	1,95	3,41	9,23E-03	1,00E+00
mt-Rnr1	0,38	1,64	2,80	9,94E-03	1,00E+00
mt-Nd1	0,24	1,20	2,19	1,05E-02	1,00E+00
mt-Cytb	0,24	1,20	2,29	1,08E-02	1,00E+00
Gm14681	0,27	1,26	2,22	1,09E-02	1,00E+00
Gm13341	0,27	1,71	3,11	1,80E-02	1,00E+00
S100a11	0,14	0,99	1,84	2,04E-02	1,00E+00
Gm29216	0,10	0,99	1,84	2,25E-02	1,00E+00
Anxa1	0,07	0,96	1,78	2,80E-02	1,00E+00
Gm28439	0,10	1,23	2,39	2,89E-02	1,00E+00
Gas5	0,07	0,79	1,50	3,08E-02	1,00E+00
mt-Nd4	0,03	0,82	1,64	3,33E-02	1,00E+00
Rps25-ps1	0,07	1,09	2,08	3,46E-02	1,00E+00
Eif3e	0,07	1,16	2,22	3,61E-02	1,00E+00
Rps15a	0,03	1,20	2,25	4,02E-02	1,00E+00
mt-Co1	0,00	0,72	1,54	4,73E-02	1,00E+00
Gm28437	0,00	1,23	2,49	4,76E-02	1,00E+00
Rps15a-ps7	-0,14	1,47	2,90	6,74E-02	1,00E+00
Rps24	-0,07	0,51	1,06	6,81E-02	1,00E+00
Gm10222	-0,14	1,37	2,77	7,19E-02	1,00E+00
Nr1d2	-0,14	0,89	1,88	8,37E-02	1,00E+00
Nudcd3	-0,14	0,89	2,19	8,61E-02	1,00E+00
9330111N05Rik	-1,64	-0,75	0,10	8,74E-02	1,00E+00
Gm12854	-0,14	0,75	1,60	9,74E-02	1,00E+00
mt-Co3	-0,20	1,02	2,49	9,76E-02	1,00E+00
Aurkb	-2,70	-1,23	0,24	1,05E-01	1,00E+00
mt-Nd4l	-0,31	1,37	3,04	1,06E-01	1,00E+00
Esd	-0,17	0,72	1,54	1,07E-01	1,00E+00
mt-Rnr2	-0,20	0,89	2,01	1,16E-01	1,00E+00
Gm43743	-0,34	1,26	2,66	1,21E-01	1,00E+00
Mrpl3	-2,49	-1,09	0,31	1,27E-01	1,00E+00

Table S5: Differential gene expression between fresh and cryopreserved MDCK cells. (MARS-Seq; top 40 genes).

Gene	Lower bound	log2 fold change	Upper bound	p-value	Adj p-value
ENSCAFG00000008021	1,24	2,15	2,99	8,84E-07	7,86E-03
ENSCAFG00000029945	0,71	1,51	2,25	7,46E-05	2,45E-01
ENSCAFG00000017688	0,94	1,95	2,95	8,25E-05	2,45E-01
ENSCAFG00000022711	0,74	1,68	2,59	3,68E-04	6,67E-01
ENSCAFG00000008336	0,97	2,18	3,26	3,75E-04	6,67E-01
ENSCAFG00000022713	0,64	1,58	2,59	9,56E-04	1,00E+00
ENSCAFG00000018174	0,50	1,31	2,12	1,01E-03	1,00E+00
ENSCAFG00000022726	0,74	1,91	3,12	1,39E-03	1,00E+00
ENSCAFG00000022729	0,77	1,88	3,32	1,40E-03	1,00E+00
ENSCAFG00000022709	0,60	1,81	3,09	2,41E-03	1,00E+00
ENSCAFG00000022717	0,44	1,51	2,65	4,46E-03	1,00E+00
ENSCAFG00000029619	0,30	1,18	2,01	6,12E-03	1,00E+00
ENSCAFG00000018187	0,27	1,07	1,85	6,66E-03	1,00E+00
ENSCAFG00000022742	0,37	1,48	2,62	8,30E-03	1,00E+00
ENSCAFG00000030258	0,34	1,38	2,32	8,67E-03	1,00E+00
ENSCAFG00000023471	0,20	0,87	1,51	9,26E-03	1,00E+00
ENSCAFG00000029710	0,37	1,58	2,82	9,31E-03	1,00E+00
ENSCAFG00000005295	0,24	1,51	2,82	1,63E-02	1,00E+00
ENSCAFG00000022730	0,37	1,88	3,02	1,63E-02	1,00E+00
ENSCAFG00000016711	0,07	0,84	1,61	3,20E-02	1,00E+00
ENSCAFG00000022728	0,10	1,38	2,69	3,27E-02	1,00E+00
ENSCAFG00000004385	-8,36	-3,49	-0,20	4,07E-02	1,00E+00
ENSCAFG00000001778	0,00	1,07	2,01	4,68E-02	1,00E+00
ENSCAFG00000007932	-2,28	-1,07	0,00	5,11E-02	1,00E+00
ENSCAFG00000008870	-1,48	-0,71	0,00	5,49E-02	1,00E+00
ENSCAFG00000015605	-0,07	1,07	2,18	5,81E-02	1,00E+00
ENSCAFG00000017066	-0,03	0,74	1,48	6,04E-02	1,00E+00
ENSCAFG00000022735	-0,03	0,97	1,81	6,48E-02	1,00E+00
ENSCAFG00000028869	-0,13	0,81	1,68	8,40E-02	1,00E+00
ENSCAFG00000007812	-0,13	0,74	1,58	8,80E-02	1,00E+00
ENSCAFG00000006810	-0,13	0,77	1,65	8,95E-02	1,00E+00
ENSCAFG00000012979	-0,13	0,71	1,48	9,35E-02	1,00E+00
ENSCAFG00000009922	-0,17	0,74	1,65	9,89E-02	1,00E+00
ENSCAFG00000010888	-2,15	-0,97	0,17	1,02E-01	1,00E+00
ENSCAFG00000007478	-2,69	-1,21	0,24	1,03E-01	1,00E+00
ENSCAFG00000013664	-3,32	-1,44	0,27	1,03E-01	1,00E+00
ENSCAFG00000016028	-0,27	1,24	2,65	1,04E-01	1,00E+00
ENSCAFG00000006747	-0,27	1,21	2,72	1,06E-01	1,00E+00
ENSCAFG00000007894	-0,24	0,94	2,05	1,06E-01	1,00E+00
ENSCAFG00000018898	-2,59	-1,18	0,24	1,08E-01	1,00E+00

Table S6: Differential gene expression between fresh and cryopreserved HEK293 cells. (SMARTseq2; top 40 genes).

Gene	Lower bound	log2 fold change	Upper boun	p-value	Adj p-value
PLAU	1,81	3,31	4,17	2,00E-08	**3,30E-04**
COLGALT2	-4,29	-3,31	-1,88	1,17E-06	**9,72E-03**
APOM	-2,42	-1,69	-1,05	2,26E-06	**1,25E-02**
NR4A3	1,53	2,83	3,94	5,09E-06	**2,11E-02**
L3MBTL1	1,43	2,58	3,56	8,07E-06	**2,41E-02**
TMEM38A	1,14	2,16	3,02	8,74E-06	**2,41E-02**
ANKRD34B	1,37	2,83	3,72	1,74E-05	**4,12E-02**
DGKH	1,53	2,93	4,07	2,08E-05	**4,31E-02**
ABCA1	1,56	3,97	5,34	3,13E-05	**5,75E-02**
RP11-348P10.2	1,40	3,05	4,01	3,67E-05	**6,08E-02**
EPHB3	1,11	3,18	4,42	4,08E-05	**6,15E-02**
RP11-338I21.1	0,79	1,72	2,45	9,50E-05	**1,31E-01**
RP11-996F15.6	0,57	1,49	2,29	1,60E-04	**2,04E-01**
STAP2	-2,19	-1,43	-0,67	2,90E-04	**3,43E-01**
WDR17	-3,59	-2,51	-1,27	3,71E-04	**4,10E-01**
ZNF416	0,99	2,29	3,50	4,72E-04	**4,89E-01**
AURKC	-2,45	-1,62	-0,64	6,60E-04	**6,43E-01**
SAMD9	-3,47	-2,23	-0,95	7,39E-04	**6,67E-01**
CTD-3001H11.2	0,48	1,30	2,07	7,72E-04	**6,67E-01**
IDNK	0,89	2,07	3,05	8,05E-04	**6,67E-01**
PLEKHA6	0,48	1,97	2,99	1,15E-03	**8,59E-01**
HSD17B14	0,67	1,88	2,83	1,16E-03	**8,59E-01**
CARD9	0,48	1,30	2,13	1,23E-03	**8,59E-01**
BSN	-3,82	-2,51	-0,99	1,27E-03	**8,59E-01**
SCN5A	0,70	1,91	2,93	1,30E-03	**8,59E-01**
CCDC120	-2,83	-1,84	-0,76	1,39E-03	**8,59E-01**
TMEM178B	0,92	2,48	4,04	1,41E-03	**8,59E-01**
HUNK	1,11	2,89	4,39	1,45E-03	**8,59E-01**
SHOX2	0,73	2,13	3,24	1,62E-03	**8,99E-01**
C14orf93	0,70	1,88	3,43	1,64E-03	**8,99E-01**
RP11-680F8.1	0,32	1,53	2,45	1,68E-03	**8,99E-01**
RP11-29B2.6	0,29	0,99	1,78	1,79E-03	**9,24E-01**
HOXA4	0,54	1,53	2,48	2,03E-03	**9,90E-01**
SATB1-AS1	0,32	1,14	2,10	2,03E-03	**9,90E-01**
EMX2	-2,10	-1,30	-0,51	2,12E-03	**1,00E+00**
CCBE1	-3,91	-2,45	-0,89	2,39E-03	**1,00E+00**
CAHM	0,45	1,34	2,16	2,47E-03	**1,00E+00**
TTLL13P	0,73	2,03	3,02	2,81E-03	**1,00E+00**
MFI2-AS1	0,29	0,86	1,62	2,94E-03	**1,00E+00**
MCF2L	0,51	1,62	2,54	3,16E-03	**1,00E+00**

Table S7: Differential gene expression between fresh and cryopreserved K562 cells. (SMARTseq2; top 40 genes).

Gene	Lower bound	log2 fold change	Upper bound	p-value	Adj p-value
HEY1	3,34	4,53	5,42	1,31E-09	**2,13E-05**
PRR16	2,48	4,26	5,29	2,36E-08	**1,93E-04**
SMIM14	2,31	3,50	4,50	1,61E-07	**7,13E-04**
ASS1	-4,66	-3,54	-2,02	1,87E-07	**7,13E-04**
MMP11	1,92	2,94	3,87	2,18E-07	**7,13E-04**
PALMD	2,25	3,67	4,56	8,37E-07	**2,28E-03**
ZNF665	1,82	3,27	4,20	1,21E-06	**2,82E-03**
FBXW8	1,55	2,94	3,87	1,72E-06	**3,52E-03**
DOCK4	1,65	3,47	4,40	4,89E-06	**8,89E-03**
FUT1	1,59	3,40	4,73	5,44E-06	**8,90E-03**
RP3-425C14	1,32	2,71	3,60	9,54E-06	**1,42E-02**
LINC00879	1,19	2,61	3,67	1,15E-05	**1,56E-02**
GRB7	1,12	2,35	3,34	1,28E-05	**1,61E-02**
ZNF726	1,92	3,24	4,20	2,13E-05	**2,49E-02**
ZNF354A	1,19	2,58	3,67	2,30E-05	**2,51E-02**
LYSMD1	1,39	2,61	3,70	3,41E-05	**3,48E-02**
FYN	1,85	3,44	4,53	4,10E-05	**3,94E-02**
PPP1R27	1,02	1,95	2,81	5,06E-05	**4,59E-02**
RAG1	1,02	3,24	4,46	5,76E-05	**4,96E-02**
CD302	-3,04	-2,02	-0,89	6,09E-05	**4,98E-02**
RP11-480I12	0,96	2,35	3,47	6,87E-05	**5,12E-02**
TCAF2	1,59	3,07	4,13	6,88E-05	**5,12E-02**
HECW2	0,96	3,37	4,40	7,77E-05	**5,52E-02**
CTC-559E9.	0,76	1,95	3,21	9,25E-05	**6,13E-02**
MCC	0,89	2,91	4,00	9,37E-05	**6,13E-02**
RP11-483I13	0,89	2,18	3,17	1,08E-04	**6,78E-02**
RHOBTB2	-3,24	-2,25	-0,96	1,30E-04	**7,85E-02**
HECA	1,65	3,50	4,73	1,63E-04	**9,53E-02**
PRG2	-6,91	-4,66	-1,55	1,79E-04	**9,89E-02**
RAB27B	1,39	4,20	5,62	1,81E-04	**9,89E-02**
INHBE	0,69	2,68	4,10	2,67E-04	**1,41E-01**
LINC01088	1,12	2,41	3,70	2,79E-04	**1,43E-01**
RP11-474G2	1,12	2,58	3,64	5,31E-04	**2,56E-01**
FIBCD1	0,53	1,45	2,35	5,40E-04	**2,56E-01**
HCFC2	1,32	3,54	4,53	5,48E-04	**2,56E-01**
RP11-1017G	0,73	2,08	3,07	6,18E-04	**2,81E-01**
TRMT44	1,09	2,68	4,00	7,17E-04	**3,17E-01**
FOXN3	1,02	2,48	3,64	8,02E-04	**3,45E-01**
AP000349.2	0,43	0,99	1,72	9,95E-04	**4,17E-01**
SAP30L	0,76	1,85	2,68	1,25E-03	**5,09E-01**

Table S8: Differential gene expression between fresh and cryopreserved PBMC. (MARS-Seq; top 40 genes).

Gene	Lower bound	log2 fold change	Upper bound	p-value	Adj p-value
RPS29	0,81	1,77	2,74	2,81E-04	**1,00E+00**
NKG7	-2,43	-1,50	-0,66	5,68E-04	**1,00E+00**
RPL9P9	0,89	2,47	6,17	2,94E-03	**1,00E+00**
GNLY	-5,74	-4,08	-0,92	7,41E-03	**1,00E+00**
CD7	-2,04	-1,08	-0,23	1,27E-02	**1,00E+00**
RPS27	0,19	1,16	2,27	1,59E-02	**1,00E+00**
RPS24	0,35	1,89	5,51	1,62E-02	**1,00E+00**
RPLP2	0,15	1,19	2,20	2,04E-02	**1,00E+00**
PFN1	-1,73	-0,92	-0,15	2,40E-02	**1,00E+00**
RPS20	0,08	1,04	1,97	2,86E-02	**1,00E+00**
CST7	-1,93	-1,00	-0,08	3,37E-02	**1,00E+00**
GZMB	-2,08	-1,08	-0,12	3,40E-02	**1,00E+00**
SPON2	-2,31	-1,12	-0,12	3,49E-02	**1,00E+00**
DAPK1	0,08	1,66	4,35	3,68E-02	**1,00E+00**
ACTB	-2,12	-1,08	-0,08	3,80E-02	**1,00E+00**
ACTG1	-1,58	-0,81	-0,08	3,86E-02	**1,00E+00**
IKZF3	-3,55	-1,62	-0,08	4,12E-02	**1,00E+00**
TYROBP	-1,97	-1,00	-0,04	4,19E-02	**1,00E+00**
CTD-3148I10.9	0,04	2,20	5,97	4,60E-02	**1,00E+00**
RPS10-NUDT3	0,00	0,77	1,50	4,65E-02	**1,00E+00**
TXNIP	-1,62	-0,81	-0,04	4,66E-02	**1,00E+00**
BIN2	-2,12	-1,04	-0,04	4,69E-02	**1,00E+00**
RPL19	0,00	0,96	1,85	4,77E-02	**1,00E+00**
PRF1	-1,81	-0,89	0,00	5,10E-02	**1,00E+00**
RPS10	-0,04	0,77	1,54	5,22E-02	**1,00E+00**
FCGR3A	-1,97	-0,96	0,00	5,34E-02	**1,00E+00**
RPL11	-0,04	1,04	2,08	5,65E-02	**1,00E+00**
TMSB4X	-2,85	-1,16	0,04	6,20E-02	**1,00E+00**
CCND3	-1,93	-0,92	0,04	6,36E-02	**1,00E+00**
B2M	-0,08	0,77	1,58	6,42E-02	**1,00E+00**
TSEN54	-6,47	-2,66	0,23	6,73E-02	**1,00E+00**
RPL26	-0,12	0,96	2,31	7,03E-02	**1,00E+00**
RP11-849F2.7	-0,12	0,96	2,31	7,03E-02	**1,00E+00**
GZMH	-1,62	-0,77	0,04	7,26E-02	**1,00E+00**
RPS19	-0,12	0,92	1,93	7,47E-02	**1,00E+00**
ADCY7	-3,97	-1,62	0,15	7,49E-02	**1,00E+00**
COMMD1	-0,23	1,85	5,66	7,64E-02	**1,00E+00**
ZNF675	-4,01	-1,66	0,19	7,71E-02	**1,00E+00**
ZNF836	-6,20	-2,47	0,27	7,73E-02	**1,00E+00**
RAC2	-1,58	-0,73	0,08	8,03E-02	**1,00E+00**

Table S9: Differential gene expression between a fresh and cryopreserved PDOX. (MARSseq; top 40 genes).

Gene	Lower bound	log2 fold change	Upper bound	p-value	Adj p-value
ZFP36	0,79	1,65	2,50	1,23E-04	**5,38E-01**
LITAF	0,75	1,61	2,40	1,34E-04	**5,38E-01**
KRT19	-2,98	-1,89	-0,93	1,36E-04	**5,38E-01**
LEFTY1	0,79	1,85	2,95	3,48E-04	**9,01E-01**
NR4A1	0,62	1,47	2,33	3,80E-04	**9,01E-01**
FOSB	0,62	1,44	2,30	5,16E-04	**9,71E-01**
GDF15	0,75	1,85	2,88	5,73E-04	**9,71E-01**
IER2	0,51	1,27	2,02	7,12E-04	**1,00E+00**
PTMAP2	-2,47	-1,54	-0,65	7,86E-04	**1,00E+00**
IL6	0,58	1,58	2,54	1,09E-03	**1,00E+00**
MDK	0,51	1,37	2,19	1,12E-03	**1,00E+00**
PTMAP5	-2,30	-1,44	-0,58	1,29E-03	**1,00E+00**
SLC35F5	0,51	1,44	2,40	1,99E-03	**1,00E+00**
TPM4	0,45	1,30	2,13	2,12E-03	**1,00E+00**
FTH1	0,38	1,58	2,71	3,11E-03	**1,00E+00**
IGFBP3	0,27	1,44	2,50	3,12E-03	**1,00E+00**
PRODH	-2,13	-1,23	-0,41	3,41E-03	**1,00E+00**
EGR1	0,31	1,10	1,82	3,99E-03	**1,00E+00**
LMO3	0,65	2,19	3,87	4,54E-03	**1,00E+00**
SOD2	0,34	1,17	1,99	4,76E-03	**1,00E+00**
TNFAIP3	0,38	1,27	2,19	5,11E-03	**1,00E+00**
UBC	0,27	1,10	1,82	6,22E-03	**1,00E+00**
BTG2	0,38	1,47	2,88	6,35E-03	**1,00E+00**
TUSC3	0,31	1,13	2,02	6,62E-03	**1,00E+00**
RIMKLB	0,45	1,68	3,05	6,84E-03	**1,00E+00**
MTMR12	0,31	1,20	2,19	7,22E-03	**1,00E+00**
RBPMS	0,24	0,99	1,75	7,74E-03	**1,00E+00**
SAT1	0,24	0,99	1,71	7,94E-03	**1,00E+00**
CALB1	-2,47	-1,41	-0,38	8,00E-03	**1,00E+00**
PTMAP4	-2,30	-1,30	-0,34	8,73E-03	**1,00E+00**
RDH10	0,27	1,17	2,13	9,52E-03	**1,00E+00**
CD81	0,21	0,96	1,71	9,56E-03	**1,00E+00**
USP43	0,24	1,10	1,95	1,06E-02	**1,00E+00**
RP11-529H20.3	-2,37	-1,30	-0,31	1,18E-02	**1,00E+00**
CD74	0,27	1,34	2,57	1,22E-02	**1,00E+00**
IL1R1	0,45	2,16	3,87	1,25E-02	**1,00E+00**
IDO2	0,48	2,13	3,87	1,27E-02	**1,00E+00**
RP11-307E17.11	-1,99	-1,10	-0,24	1,31E-02	**1,00E+00**
RPS11	0,17	0,86	1,51	1,32E-02	**1,00E+00**
TXNRD1	0,17	0,89	1,58	1,33E-02	**1,00E+00**
